# Huntingtin Interacting Proteins Are Genetic Modifiers of Neurodegeneration 

**DOI:** 10.1371/journal.pgen.0030082

**Published:** 2007-05-11

**Authors:** Linda S Kaltenbach, Eliana Romero, Robert R Becklin, Rakesh Chettier, Russell Bell, Amit Phansalkar, Andrew Strand, Cameron Torcassi, Justin Savage, Anthony Hurlburt, Guang-Ho Cha, Lubna Ukani, Cindy Lou Chepanoske, Yuejun Zhen, Sudhir Sahasrabudhe, James Olson, Cornelia Kurschner, Lisa M Ellerby, John M Peltier, Juan Botas, Robert E Hughes

**Affiliations:** 1 Prolexys Pharmaceuticals, Salt Lake City, Utah, United States of America; 2 Department of Molecular and Human Genetics, Baylor College of Medicine, Houston, Texas, United States of America; 3 Clinical Research Division, Fred Hutchinson Cancer Research Center, Seattle, Washington, United States of America; 4 Buck Institute for Age Research, Novato, California, United States of America; University of Minnesota, United States of America

## Abstract

Huntington's disease (HD) is a fatal neurodegenerative condition caused by expansion of the polyglutamine tract in the huntingtin (Htt) protein. Neuronal toxicity in HD is thought to be, at least in part, a consequence of protein interactions involving mutant Htt. We therefore hypothesized that genetic modifiers of HD neurodegeneration should be enriched among Htt protein interactors. To test this idea, we identified a comprehensive set of Htt interactors using two complementary approaches: high-throughput yeast two-hybrid screening and affinity pull down followed by mass spectrometry. This effort led to the identification of 234 high-confidence Htt-associated proteins, 104 of which were found with the yeast method and 130 with the pull downs. We then tested an arbitrary set of 60 genes encoding interacting proteins for their ability to behave as genetic modifiers of neurodegeneration in a *Drosophila* model of HD. This high-content validation assay showed that 27 of 60 orthologs tested were high-confidence genetic modifiers, as modification was observed with more than one allele. The 45% hit rate for genetic modifiers seen among the interactors is an order of magnitude higher than the 1%–4% typically observed in unbiased genetic screens. Genetic modifiers were similarly represented among proteins discovered using yeast two-hybrid and pull-down/mass spectrometry methods, supporting the notion that these complementary technologies are equally useful in identifying biologically relevant proteins. Interacting proteins confirmed as modifiers of the neurodegeneration phenotype represent a diverse array of biological functions, including synaptic transmission, cytoskeletal organization, signal transduction, and transcription. Among the modifiers were 17 loss-of-function suppressors of neurodegeneration, which can be considered potential targets for therapeutic intervention. Finally, we show that seven interacting proteins from among 11 tested were able to co-immunoprecipitate with full-length Htt from mouse brain. These studies demonstrate that high-throughput screening for protein interactions combined with genetic validation in a model organism is a powerful approach for identifying novel candidate modifiers of polyglutamine toxicity.

## Author Summary

Huntington's Disease (HD) is a fatal inherited neurodegenerative disease, which typically begins in middle age and progresses with symptoms of severe uncontrolled movements and cognitive dysfunction. HD is uniformly fatal with death occurring ten to 15 years after onset of symptoms. There is currently no effective treatment for HD. The genetic mutation underlying HD causes a protein called huntingtin (Htt) to contain an abnormally long tract of the amino acid glutamine. This extended span of glutamines changes the shape of the Htt protein, which can cause it to interact in abnormal ways with other cellular proteins. In this study, we have identified a large number of new proteins that bind to normal and mutant forms of the Htt protein. To establish a potential role for these interacting proteins in HD, we show that changing the expression of many of these proteins can modulate the pathological effects of mutant Htt on fly neurons that deteriorate when they express mutant Htt. Identifying cellular proteins that bind to Htt and modulate its pathological activity may facilitate the discovery of an effective treatment for HD.

## Introduction

Huntington's Disease (HD) is a member of a family of dominantly inherited neurodegenerative diseases caused by expansion in a glutamine-encoding CAG tract. HD occurs when the polyglutamine (polyQ) tract in huntingtin (Htt) expands beyond ~35 glutamine (Q) repeats and manifests with movement disorder, psychological disturbances, and cognitive dysfunction progressing over a period of about ten to 15 years until death. Currently there is no effective treatment or cure for HD.

Mutant Htt is thought to cause cellular dysfunction, neurodegeneration, and associated clinical features primarily through a toxic gain of function [1]. Indeed, proteins containing expanded polyQ tracts are toxic when expressed in a wide range of experimental transgenic systems including yeast, cultured mammalian cells, Caenorhabditis elegans*, Drosophila,* and mouse [2–4]. Determining the precise mechanism of polyQ-mediated toxicity is a subject of intense inquiry, and there is evidence supporting a role for aberrant protein-protein interactions in pathogenesis. In HD, expanded Htt is processed to N-terminal fragments that form inclusions found both in the cytoplasm and nucleus [5,6]. A number of proteins localize to expanded polyQ inclusions, including ubiquitin/proteasome components, heat shock proteins, and transcription factors [7–12]. These findings support the idea that mutant Htt may interfere with the functions of diverse cellular proteins directly, through protein interactions. Some interacting proteins have been shown to be functionally compromised when bound to mutant Htt [11,13,14]. In addition, some of these proteins localize to insoluble Htt-fragment-containing inclusions present in affected tissues [15,16]. Recent work, however, has suggested that inclusions may be benign or even protective and that other misfolded forms of Htt may be the primary toxic species [17–19]. Since interactions between cellular proteins and soluble or aggregated Htt may have a general role in HD pathogenesis, identification of Htt-interacting proteins will further elucidate toxic mechanisms and therapeutic targets for the disease.

Htt is a large, ubiquitously expressed protein comprised nearly entirely of HEAT repeats, a characteristic protein-protein interaction motif [20,21]. Nearly 50 proteins capable of interacting directly with Htt or Htt fragments have been described. Most proteins have been found to interact with N-terminal polyQ containing Htt fragments and, in some cases, the strength of these interactions has been shown to be sensitive to the length of the polyQ tract [22,23]. Htt-interacting proteins represent diverse cellular roles including intracellular transport, transcription, and ubiquitin-mediated proteolysis. These observations suggest that the normal function of Htt involves multiple protein-protein interactions in the context of diverse multiprotein cellular complexes. Indeed, loss of normal Htt function is a component of HD pathology [24]. Identifying Htt protein-protein interactions may help to elucidate the functions of wild-type Htt as well as the novel gain of function of mutant Htt.

In this study we report a large set of novel Htt-fragment-interacting proteins using yeast two-hybrid (Y2H) and affinity pull-down/mass spectrometry (MS) protein interaction screens. We used both approaches in parallel in an effort to define a comprehensive set of interactors, as there is evidence that each method explores different groups of interacting proteins [25–28]. We reasoned that if expanded Htt can influence the functions of its interacting proteins (and vice versa), genes encoding interacting proteins should be enriched for genetic modifiers of neurotoxicity mediated by expression of a mutant Htt fragment. We used a *Drosophila* model of polyQ toxicity to test this idea and found that 45% of the interactors behave as high-confidence genetic modifiers (i.e., interaction confirmed with more than one allele). Importantly, protein interactions validated as genetic modifiers in *Drosophila* were equally represented in the Y2H and MS derived datasets, demonstrating the complementary nature of these independent methods.

A standard method for validation of large-scale interaction datasets relies on co-affinity precipitation of samples of the protein interaction pairs [29,30]. Whereas co-affinity precipitation does confirm a physical interaction, it does not establish the biological relevance of that interaction. The high-content validation method used in this study (genetic interaction in a whole organism) strongly supports the conclusion that this dataset is highly enriched for interacting proteins with functional roles in polyQ-mediated neurodegeneration. Using co-immunoprecipitation, we show further that a number of these modifier proteins physically associate with Htt in brain tissue of transgenic mice expressing full length Htt protein.

An ultimate result of this study is to provide insight into potential therapeutic targets for HD. The 17 loss-of-function suppressors of *Drosophila* HD reported here constitute a significant collection of novel targets (and pathways) to be considered as targets for therapeutic intervention.

## Results

### Identification of Novel Htt-Fragment-Interacting Proteins

**Figure 1:**
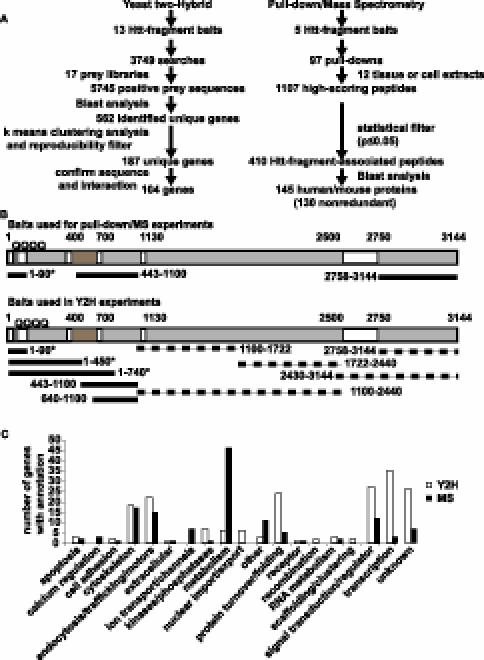
Results of the Physical Interaction Screens (A) Overview of the discovery workflow is represented. Y2H and pull-down/MS workflows are shown on the left and right, respectively. The number of Htt-fragment baits used for Y2H searches or pull downs includes wild-type (23 Q) and mutant (48 Q and 55 Q) forms. Not all Htt fragments were successfully expressed in bacteria or yielded positive interactions in Y2H screens. For Y2H positive prey identification, the top blast score was chosen. The total number of genes found in pull down/MS includes 15 mouse and human homologs; the nonredundant set does not include mouse homologs (see Supporting Information). (B) A diagram of Htt baits used in Y2H and MS experiments is presented. Structural features (HEAT repeat domains and protease cleavage region) are indicated by shaded boxes on a diagram of the Htt protein. Numbers indicate reference amino acids positions (with respect to NP_002102). Lines representing Htt-fragment baits with associated amino acid positions indicated by numbers are shown relative to the diagram of Htt. We purified three Htt-fragments (top panel) from bacteria in sufficient quantities for pull-down/MS experiments. Htt-fragment baits used in Y2H screens are shown on the bottom panel. Some baits did not yield positive interactions (dotted lines). Htt clones that contained the polyQ sequence were generated in wild-type (23 Q) and expanded (55 Q, 75 Q, and 97 Q) forms (asterisk). (C) A functional analysis of Htt-fragment-interacting proteins is presented. The number of proteins representing the indicated functional categories found in Htt-fragment Y2H screens (white bars) or pull down/MS (black bars) are shown. Proteins were assigned to categories based on gene ontology. Only categories with more than one protein assigned are shown.

In a comprehensive search for novel Htt-fragment-interacting proteins, we performed two large-scale screens for interactions using MS and Y2H methods (Figure 1A). Multiple fragments of Htt, including both wild-type and mutant N-terminal fragments, were cloned and expressed for pull-down experiments and Y2H screens (Figures 1B and S1). We purified five recombinant Htt-fragment baits (corresponding to amino acid residues 1-90-23 Q, 1-90-48 Q, 1-90-75 Q, 443–1,100, and 2,758–3,114) from Escherichia coli in sufficient quantities for pull-down experiments. A total of 97 pull-down experiments were performed with these Htt-fragment baits and mammalian tissue or cell protein lysates (Figure 1). Tandem affinity purification (TAP)-tagged Htt-fragment containing protein complexes were allowed to form in protein extracts prepared from mouse or human brain tissue or mouse muscle tissue. Complexes were copurified with the affinity tagged Htt-fragment proteins and analyzed by MS. Of the five Htt-fragment bait proteins, only the 1–90 amino terminal fragments yielded specific and reproducible protein complexes. The wild-type and mutant Htt-fragment baits (corresponding to exon 1 of the HD gene) (Figure 1B) were also used to probe protein extracts prepared from cultured cells (HEK293, HeLa, and M17 neuroblastoma). Using the database-searching tool MASCOT, we generated a primary dataset of 1,107 unique high-scoring peptides present in the Htt-fragment pull downs (Figure 1A; Table S1). To generate a high-confidence interaction list, we subjected these peptides to a statistical test for specific association with Htt fragments by comparison to a database of 15,131 high-scoring peptides identified in pull downs performed with 88 different protein baits (unpublished data). This analysis was used to generate a *p*-value for the association of a particular peptide with Htt-fragment pull downs. A total of 410 unique peptides from Htt-fragment pull downs met a *p*-value limit of ≤0.05, and each of these peptides was manually validated by inspection of the MS spectra (see detailed methods in Supporting Information). The data were filtered further by excluding any proteins identified by peptides observed in control pull downs with TAP-tag alone or proteins containing any peptides not meeting the *p*-value cut-off (i.e., peptides not specific to Htt-fragment pull downs). These methods identified 145 mouse and human proteins specific to Htt-fragment pull downs and eliminated many proteins considered to be false positives in other studies (Tables 1 and S1) [25]. Genes encoding orthologs of 28 of these proteins were tested for genetic interaction with a truncated mutant N-terminal human HD gene in a *Drosophila* model of polyQ toxicity.

**Table 1:**
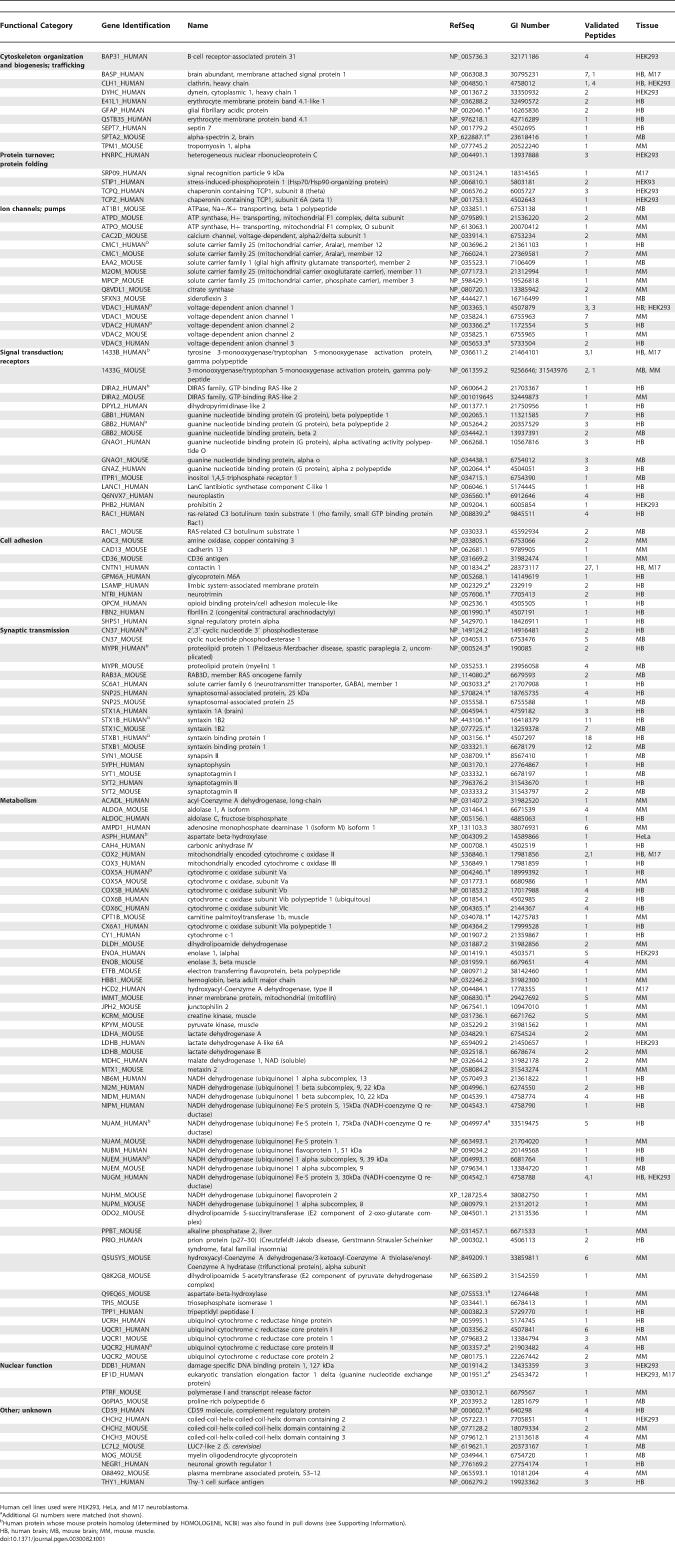
Selected Proteins Identified in Pull Downs with Htt Fragments

In addition to solution-based MS protein interaction studies, we performed Y2H searches with Htt-fragment baits using a high-throughput automated screening platform [31]. A total of 3,749 individual Y2H searches of HD fragment baits were performed against prey libraries prepared from 17 different human tissue cDNA sources (Figure 1). Multiple overlapping baits were searched extensively but only baits located near the Htt N terminus (including polyQ containing fragments) gave reproducible interactions (Figure 1B, solid lines). PolyQ containing Htt-fragment baits of amino acids 1–90, 1–450 or 1–740 were screened in both wild-type (23 Q) and mutant (>45 Q) forms. Screens were performed under stringent selection conditions requiring simultaneous activation of two independent auxotrophic reporter genes, *HIS3* and *ADE2* [31,32]. Initial results identified a total of 562 unique interacting prey proteins (Figure 1A; Tables S2 and S3). Because Y2H screens have been estimated to contain up to 50% false positives among the primary positives [33,34], the data were filtered using stringent criteria to eliminate false positives and generate a high-confidence dataset. First, only interactions that had been independently observed at least three times in Y2H screens were included. Next, proteins were excluded if they were observed to interact with more than 174 unique partners in a database of 110,000 interacting protein pairs generated from approximately 290,000 Y2H screens. These searches were performed in a large random screen for human protein interactions (unpublished data). This cut-off, designed to eliminate promiscuous interactors, was calculated by k-means clustering analysis of the random dataset [31]. Finally, genes encoding interacting preys were recovered from positive yeast colonies, sequenced twice, and reintroduced with Htt-fragment bait into naive Y2H assay cells. Genes were excluded if the interaction with Htt fragments could not be reproduced in the Y2H assay (as measured by activation of two reporter genes). Previously published Htt and Htt-fragment interactors were included in the final list regardless of the exclusion criteria. A total of 104 unique interacting proteins (18% of the primary dataset) met these conditions and were included in the final high-confidence dataset (Table 2). A complete list of all Htt-fragment interactions found in our Y2H screens is shown in Table S2. Sequences derived from all positive colonies used to identify the interacting proteins are presented in Table S3. Orthologs of 35 of these genes were tested for genetic interactions with a mutant human N-terminal portion of the HD gene in a *Drosophila* model of polyQ toxicity.

**Table 2:**
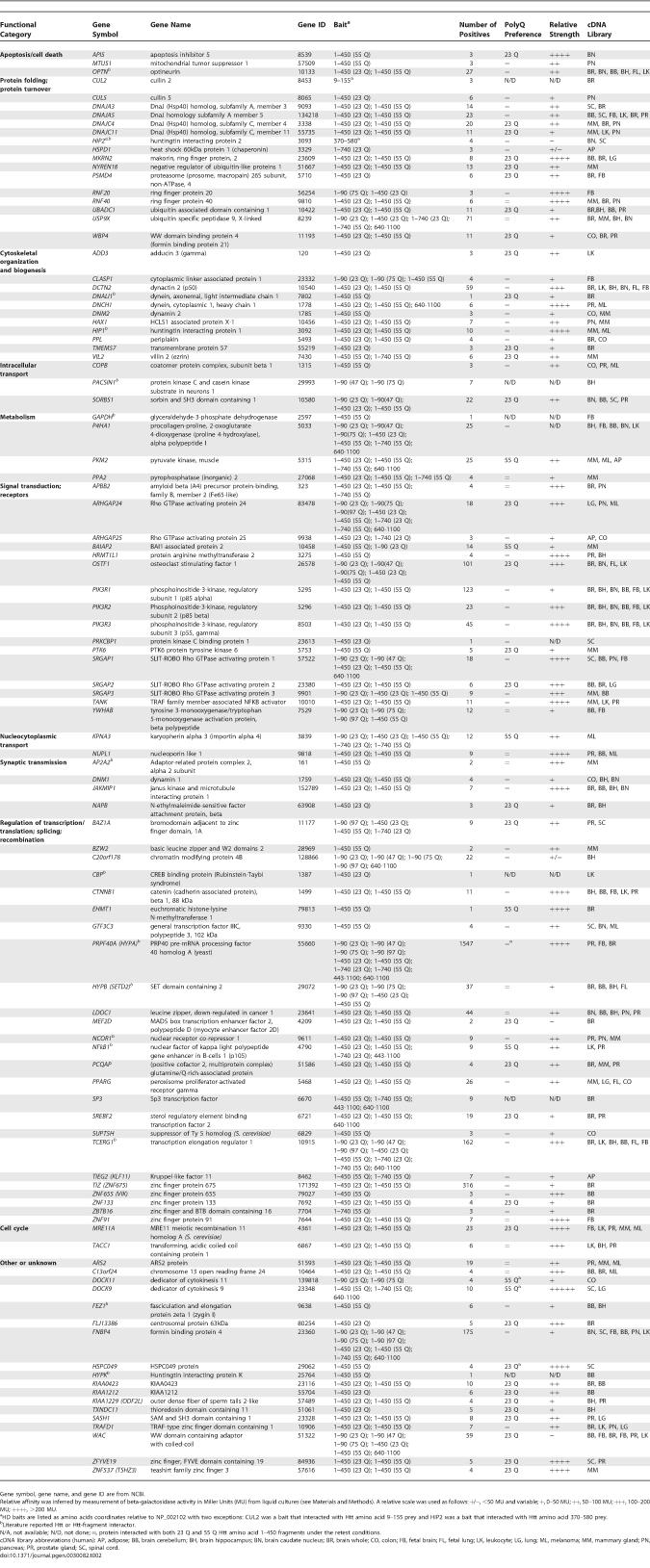
Selected Proteins Interacting with Htt Fragments in a Y2H Assay

While more than 3,500 searches were performed with Htt fragments, after 800 searches the rate of discovery for novel interacting proteins approached zero, indicating that these screens were close to saturation (Figure S2). This result demonstrates that the total number of Htt-fragment-interacting proteins discovered in our Y2H screens represents a finite set and is not simply a function of the number of searches.

Examining the gene ontology annotations associated with interacting proteins reveals that the two methods differ to some degree in the type of proteins identified (Figure 1C). Y2H clearly identified more proteins involved in protein turnover, signal transduction, and transcription, while MS identified more proteins involved in metabolic processes. However, proteins involved in cytoskeletal or protein-trafficking processes were similarly represented among the Y2H and MS data. Overall, there was little overlap of specific interacting proteins between the two datasets. Only four high-confidence proteins were found using both methods: clathrin, pyruvate kinase, GAPDH, and YWHAB (Tables S1 and S2). Two of these, clathrin and GAPDH have been previously reported to associate with Htt fragments [35,36].

To directly address the biological relevance of the Htt-fragment protein-interaction dataset and to assess the relative validity of results generated using the Y2H and MS methods, we tested a sample of interacting proteins in a high-content independent method, a genetic modifier assay in a fly model of polyQ toxicity.

### Validation of Htt-Fragment-Interacting Proteins in a *Drosophila* Model of PolyQ Toxicity

An arbitrary sample of 60 proteins in the dataset was tested for the ability to modify an Htt-fragment-induced neurodegeneration phenotype in *Drosophila*. This polyQ toxicity model was generated using an N-terminal fragment of the human *HD* cDNA, encoding the first 336 amino acids of the protein, including a 128 Q expansion in exon 1 (see Materials and Methods). Directed expression of this expanded human HD transgene fragment in the *Drosophila* eye causes a neurodegenerative phenotype evident by external examination and retinal histology. Of the 234 nonredundant mammalian protein interactors found in the MS and Y2H screens, 213 had apparent orthologs in *Drosophila* (unidirectional top hit with BLAST score less than 10^−3^), and 127 of these had available *Drosophila* stocks suitable for screening. We tested 60 of these, divided roughly equally between genes discovered using Y2H (35) and MS (28) methods (including three genes found in common), for possible genetic interactions in the fly model of polyQ toxicity (Table S4). A total of 48 of the 60 genes in the sample (80%) either enhanced or suppressed the expanded Htt-fragment-induced neurodegeneration in the *Drosophila* eye when tested in either over-expressing or in partial loss-of-function strains (Tables 3 and S5). In some cases a modifier effect was observed, but only one background strain could be tested (Table S5). However, for 27 of these genes, modification either by more than one allele or in more than one genetic background was observed. These genes comprise a high-confidence set of genetic modifiers of mutant Htt-fragment toxicity (Figures S3 and S4; Table 3). The 27 high-confidence modifiers represent a 45% validation rate among those interactors tested. Since the collection of genes tested in the fly assay represented an arbitrary sample of the protein interaction collection, this result indicates that as much as half of the proteins in our dataset may be modifiers of mutant Htt toxicity. The hit rate for genetic modifiers seen among our interactors is an order of magnitude higher than the expected 1%–4% typically observed in unbiased genetic screens [37–39], including a comparable modifier screen using a *Drosophila* model of the polyQ disease spinocerebellar ataxia type 1 [40]. Validation rates for proteins discovered by either Y2H (27/35 or 77%) or MS (21/28 or 75%) methods were similar, indicating that these methods are comparable in their ability to uncover biologically relevant interactions (Table 3). These relative validation rates demonstrate further that the MS and Y2H datasets are complementary in nature and that each dataset is similarly enriched for genes and proteins that modify mutant Htt toxicity in vivo. Furthermore, the majority of these modifiers were discovered in interaction screens performed with human brain protein extracts or brain-derived cDNA libraries indicating that they are expressed in tissues relevant to HD (Tables 1 and 2).

**Table 3:**
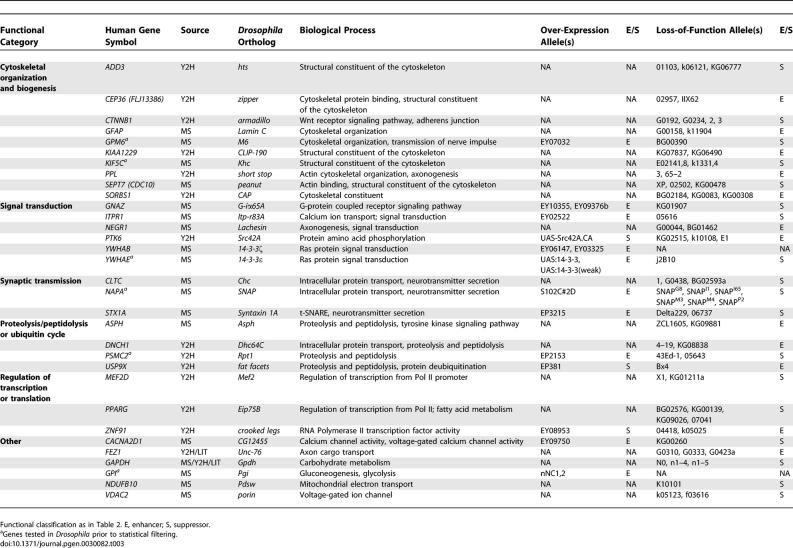
Modifiers of the Expanded Htt-Fragment-Induced Eye Phenotype

**Figure 2:**
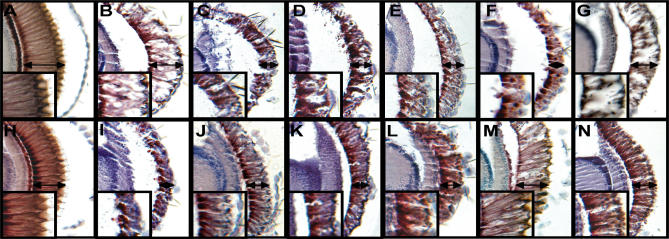
Modification of the Phenotypes Caused by N-Terminal Expanded Htt in the *Drosophila* Eye Retinal sections of adult *Drosophila* eyes show modification of the phenotypes caused by expression of different levels (B and I) of a transgene encoding an N-terminal expanded Htt fragment. Enhancers (C–G) and suppressors (J–N) include proteins involved in cytoskeletal organization (C) and (J), signal transduction (D) and (K), neurotransmitter secretion (E) and (L), proteolysis/peptidolysis and the ubiquitin cycle (F) and (M), and transcriptional/translational regulation (G) and (N). Retinal sections of day 5 control flies cultured at 25 °C expressing the gene that encodes expanded N-terminal Htt fragment *(GMR-GAL4*/+; *UAS:128Qhtt[M64]*/*+)* (B) show a degenerative phenotype when compared to controls of the same age and cultured at the same temperature *(GMR-GAL4*/+*)* (A). The phenotype consists of a shortening (see arrow) and detachment of the retina, as well as the presence of vacuoles in the retina. The Htt-fragment-induced phenotype can be enhanced by (C) reduced levels of zipper *(GMR-GAL4/P{PZ}zip^02957^; UAS:128Qhtt[M64]/+*), (D) reduced levels of Src oncogene at 42A *(GMR-GAL4*/P{lacW}Src42A^k10108^; *UAS:128Qhtt[M64]*/*+),* (E) overexpression of soluble N-ethylmaleimide-sensitive -attachment protein *(GMR-GAL4*/+; *UAS:128Qhtt[M64]*/UAS-S102C#2D*),* (F) reduced levels of fat facets *(GMR-GAL4*/+; *UAS:128Qhtt[M64]*/faf^Bx4^ *),* and (G) reduced levels of crooked legs *(GMR-GAL4*/P{PZ}crol^04418^cn^1^; *UAS:128Qhtt[M64]*/*+)*. None of these mutations cause an abnormal eye phenotype in flies carrying the *GMR-GAL4* driver but not the *UAS:128Qhtt[M64]* transgene (unpublished data). However, when combined with an N-terminal expanded Htt fragment, they lead to an even larger decrease in retinal thickness sometimes accompanied by an increase in retinal detachment and vacuolization. Retinal sections of day 1 control flies cultured at 27 °C expressing a gene that encodes an expanded N-terminal Htt fragment *(GMR-GAL4*/+; *UAS:128Qhtt[M64]*/*+)* (I) show a severe degenerative phenotype when compared to GMR controls of the same age and cultured at the same temperature (H). The phenotype consists of a shortening (see arrow) and detachment of the retina, as well as the presence of vacuoles in the retina. The Htt-fragment-induced phenotype can be suppressed by (J) reduced levels of hu li tai shao *(GMR-GAL4*/P{lacW}hts^k06121^; *UAS:128Qhtt[M64]*/*+),* (K) reduced levels of G protein iαsubunit 65A *(GMR-GAL4*/; *UAS:128Qhtt[M64]*/P{SUPor-P}G-ia65A^KG01907^ry^506^ *),* (L) reduced levels of clathrin heavy chain *(*Chc^1^/+ *GMR-GAL4*/+; *UAS:128Qhtt[M64]*/*+),* (M) reduced levels of Rpt1 *(GMR-GAL4*/P{PZ}Rpt1^05643^cn^1^; *UAS:128Qhtt[M64]*/*+),* and (N) reduced levels of myocyte enhancing factor 2 *(GMR-GAL4*/Df(2R)X1,Mef2[X1]; *UAS:128Qhtt[M64]*/*+)*. These mutations decrease the vacuolization and increase the retinal thickness as well as virtually eliminating the retinal detachment.

Among the 27 high-confidence modifiers, partial loss-of-function mutations were tested for 27 of them and over-expression mutations for nine. A total of 18 of the modifiers behaved as suppressors of neurodegeneration, (14 by partial loss-of-function and four by over-expression) (Figure S3), whereas 18 behaved as enhancers (13 by partial loss-of-function and five by over-expression) (Figure S4). In all 13 cases where both over-expression and loss-of-function alleles were tested, suppression was observed in one condition and enhancement in the other. These modifiers cluster into several functional groups including proteins involved in cytoskeletal organization and biogenesis, signal transduction, synaptic transmission, proteolysis, and regulation of transcription or translation (Table 3). Histological analysis of eye phenotypes from representative enhancers and suppressors from each of these groups is shown (Figure 2).

One interesting subset of modifiers is a group of proteins involved in SNARE-mediated vesicle fusion [41,42]. This includes STX1A, NAPA, and the voltage-gated calcium channel delta subunit CACNA2D1. Interestingly, alleles encoding all of these proteins act both as loss-of-function suppressors and gain-of-function enhancers in the fly assay. Collectively, these modifier results point toward a model of Htt toxicity involving dysregulation in synaptic function at the level of SNARE-mediated vesicle fusion.

**Figure 3:**
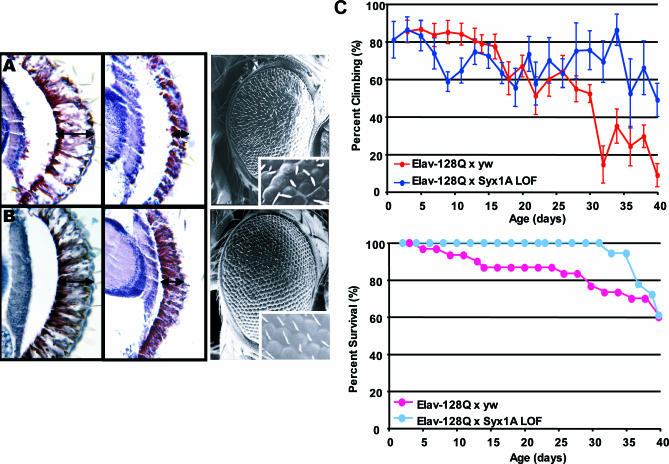
Modification of the Expanded Htt-Fragment-Induced Phenotype by a STX1A Loss-of-Function Mutation Modification was observed both in the eye (external phenotype and retinal sections) and in the nervous system (climbing ability and survival). (A) Retinal sections of day 5 flies raised at 25 °C (left), day 1 flies raised at 27 °C (middle), and standard error of mean of day 5 flies raised at 29 °C (right) expressing a gene that encodes expanded N-terminal Htt fragment *(GMR-GAL4/+*; *UAS:128Qhtt[M64]/+)*. (B) Retinal sections of day 5 flies raised at 25 °C (left), day 1 flies raised at 27 °C (middle), and standard error of mean of day 5 flies raised at 29 °C (right) expressing a gene that encodes expanded N-terminal Htt fragment and carrying reduced levels of STX1A *(GMR-GAL4/+; UAS:128Qhtt[M64]/Syx1A229ry506)*. Note suppression of both the retinal and external eye phenotypes at all three temperatures. Overexpression of STX1A shows enhancement of the retinal degeneration and external 128 Qhtt phenotype (unpublished data). (C) Climbing assay (top) and survival assay (bottom) results confirm the suppression observed in the eye assay. Shown in red/pink are the climbing performance and survival curve of a population of females flies expressing a gene that encodes expanded N-terminal expanded Htt fragment *(elav-GAL4/+; UAS:128Qhtt[F27B]/+)*. Shown in blue/light blue are the improved climbing performance and survival curve of a population of females flies expressing a gene that encodes expanded N-terminal Htt fragment and carrying a heterozygous loss-of-function mutation in STX1A *(elav-GAL4/+; +/+; UAS:128Qhtt[F27B]/Syx1A229 ry506)*. (x-Axis, age of flies in days; y-axis, percent surviving or climbing flies; LOF, loss-of-function).

Additional experiments were performed to further validate a role for a SNARE component in modifying mutant Htt toxicity (Figure 3). In contrast to expression in the eye, pan-neural expression of N-terminal expanded Htt leads to a shortened lifespan in the fly model of polyQ toxicity. Pan-neural expression also results in late-onset progressive motor dysfunction that can be quantified in terms of climbing performance as a function of age. These behavioral assays confirm the results obtained in the eye assay: partial loss-of-function of STX1A ameliorates both the disorganization and fusion of ommatidia seen in flies expressing the gene that encodes N-terminal expanded Htt as well as the retinal degeneration. The shortened life-span and the late-onset progressive motor dysfunction phenotypes were also improved by a partial loss-of-function of STX1A, confirming that the modifier effects seen in the eye were not limited to a particular phenotypic assay (Figure 3B and 3C). Htt is known to interact with proteins involved in endocytosis and vesicle trafficking such as PACSIN1, HAP1, HIP1, and HIP14 [22], however, this is the first report showing that Htt interacts directly with the SNARE complex and that partial loss-of-function can suppress mutant Htt toxicity.

**Figure 4:**
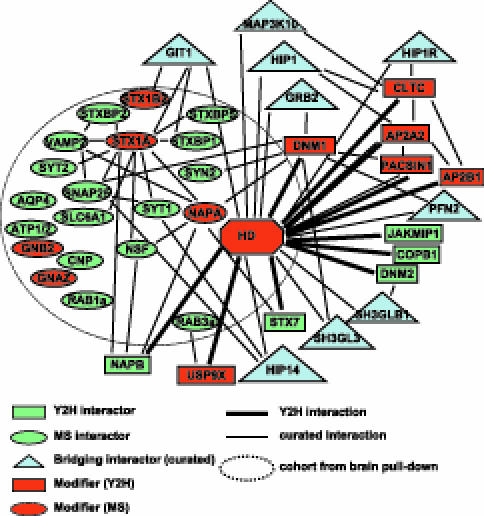
A Network of Protein Interactions Involved in Vesicle Traffic A network is shown that includes protein interactions described in this study and interactions curated from the public domain (NCBI Entrez Gene). Htt-fragment-interacting proteinss found in this study are indicated as ovals (MS) or rectangles (Y2H). Binary Y2H interactions found in this study are indicated as thick lines. Proteins contained in the dotted circle were identified in Htt-fragment pull downs using brain lysates. Thin lines indicate curated protein interactions. Curated bridging interactions (blue triangles) are defined as proteins reported to interact with HD and at least one other protein in the network. Proteins whose *Drosophila* ortholog genes acted as modifiers in this study are indicated in red.

A network summarizing interactions relevant to Htt and proteins with gene ontology annotations (http://www.geneontology.org) related to vesicle traffic and/or neurotransmission is shown in Figure 4. Included here are Y2H interactions (rectangles and thick lines) and proteins identified by MS (ovals) in pull downs using lysates prepared from mouse and/or human brain tissue (set included in dotted circle). A total of 11 proteins in this interaction subnetwork (shown in red) are encoded by human orthologs of genes shown to act as modifiers in the *Drosophila* model of polyQ toxicity (Tables 3 and S3). Notably, several modifiers are present in a highly connected cluster of Htt-fragment-interacting proteins known to function in receptor-mediated endocytosis: CLTC, AP2A2, AP2B1, PACSIN1, and DNM1. The observations that Htt is localized to endosomal vesicles and associated with clathrin in fibroblasts derived from HD patients [5,43] and that vesicle associated proteins are found in Htt-fragment inclusions [44] makes this interconnected cluster of modifiers particularly striking. Curated Htt-fragment-interacting proteins obtained from BioGRID (http://www.thebiogrid.org) [45] and/or the Human Protein Reference Database (http://www.hprd.org) [46,47] are included in the network. These bridging proteins (blue triangles) represent all curated interactions contained in these databases that connect HD to at least one other protein in the subnetwork though a single protein node and link some of our novel Y2H interactions and MS associations to known Htt-interacting proteins (e.g., HIP1, GIT1). Together, this interaction network provides additional proof that our dataset is enriched for proteins that are important in HD pathogenesis and underscores the role of proteins involved in vesicle traffic as being relevant to HD function and pathology.

### Validation of Htt-Fragment-Interacting Proteins Using Immunoprecipitation

**Figure 5:**
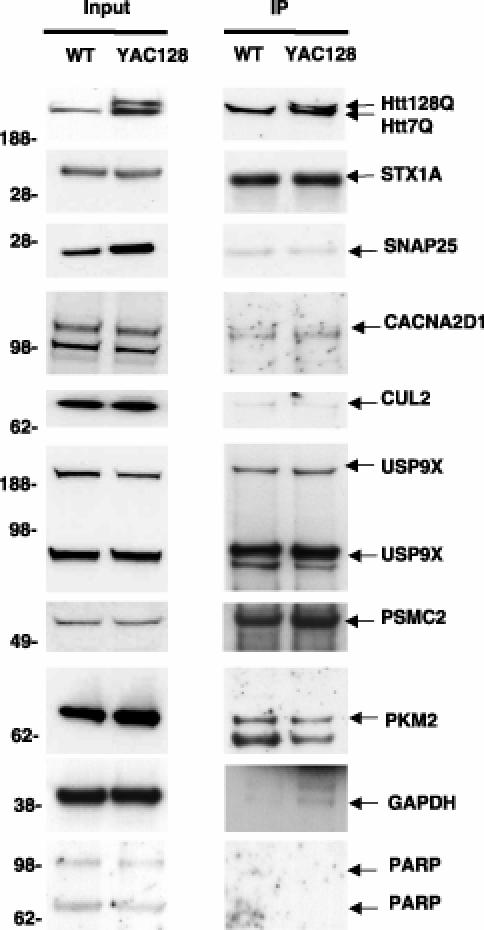
Co-Immunoprecipitation of Huntingtin-Interacting Proteins from YAC128 Mouse Brain Htt was immunoprecipitated with mouse monoclonal Htt antibody and probed with rabbit polyclonal Htt BKP1 antibody (top right panel). The input for each protein (left panels) and resulting immunoprecipitation are shown (right panels). The lower molecular weight band in the PKM2 immunoprecipitation is an immunoglobulin (IgG) band. GAPDH is included as a positive control. PARP is included as a negative control.

For further in vivo validation of Htt-fragment protein interactions in mammalian tissue, we performed co-immunoprecipitation experiments from brains of wild-type mice and mice expressing a 128 Q full length YAC transgene [48]. Figure 5 shows the results of co-immunoprecipitation experiments using antibodies raised against Htt-fragment-interacting proteins. In all, we observed co-immunoprecipitation with seven of 11 interacting proteins tested. These included the SNARE-associated proteins STX1A and CACNA2D1, both of which are modifiers in the *Drosophila* assay. We also observed co-immunoprecipitation with SNAP25 (another SNARE component). Other modifiers observed to associate with Htt in mouse brain were the ubiquitin hydrolase USP9X and the proteasome component PSMC2. None of these interacting proteins appeared to show a strong preference for wild-type versus CAG expanded Htt in this assay. Immunoprecipitation using antibodies directed against GAPDH and PARP are included as positive and negative controls. We observed a polyQ length-dependent association of GAPDH with Htt. The GAPDH protein has been reported to bind Htt and act as a modifier of mutant Htt toxicity [36,49].

Overall, in this sample, we observed a 60% validation rate in this assay (seven of 11 proteins tested). Of the seven proteins observed to co-immunoprecipitate with Htt from mouse brain, three were discovered using Y2H (CUL2, PSMC2, and USP9X), two were discovered using MS (STX1A and SNAP25), and one by both methods (PKM2). This further underscores a specific utility of both methods for discovery of interacting proteins.

## Discussion

Although the gene encoding Htt was identified over a decade ago, the normal function of this protein and the precise mechanisms by which expanded polyQ exerts its toxic effects remain the subjects of intense inquiry. In this study we identified 234 potential new Htt-associated proteins using high-throughput proteomic screens. The diverse functions of Htt and Htt-fragment protein partners and modifiers reported here are consistent with the functional diversity of pathogenic processes and targets in HD. Htt is localized to a number of different cellular compartments, and there is a large body of evidence showing that mutant Htt fragments can interfere with a diverse range of proteins and pathways including, transcriptional activation and co-activation [12,13,15], ubiquitin-mediated proteolysis [50], mitochondrial energy metabolism [51,52], receptor-mediated signal transduction [53], axonal transport [54], and vesicle trafficking [43,44]. These observations suggest models of Htt-mediated pathology that involve interference in multiple cellular pathways.

Furthermore, we have identified a novel association between Htt fragment and components of the vesicle secretion apparatus (Table 1). Stx1A, NAPA, and CACNA2D1 were confirmed as modifiers in the fly polyQ toxicity model (Table 3), and SNAP25, STX1a, and CACNA2D1 proteins were observed to co-immunoprecipitate with full length Htt from mouse brain (Figure 5). Protein interactions and localization experiments have placed Htt primarily at postsynaptic sites (reviewed in [55]), but Htt has also been shown to be associated with N-type calcium channels in presynaptic cells [56]. These results suggest that modulation of SNARE-mediated neurotransmitter secretion may be a normal function for Htt and/or may be perturbed by mutant Htt.

In addition to the general large-scale protein interaction screens reported for human proteins, two screens have been reported that focus specifically on proteins related to polyQ disease. A large-scale Y2H screen for Htt-fragment binding proteins uncovered 15 novel interacting proteins, including GIT1, an enhancer of polyQ aggregation [57]. A more recent screen for protein interactions relevant to inherited ataxias reported a large network of interaction involving 54 proteins implicated in human ataxia [29]. Interestingly, there was more overlap between high-confidence interactions in our dataset and the previously published Htt dataset [57] than the ataxia dataset [29], suggesting that protein-protein interactions may contribute to pathogenic specificity found among the polyQ diseases. Validation of interactions in the ataxin network study relied on demonstration of co-affinity precipitation of tagged expressed protein pairs. Here we tested the ability of a genetic model to validate protein interactions. 48 of 60 genes tested in a polyQ-induced fly eye degeneration model of HD modified the polyQ-induced toxicity, suggesting that this list contains protein interactors that also genetically interact with Htt. Our validation rate using the *Drosophila* genetic model (80%) is similar to that found using co-affinity purification in the ataxia and Htt studies (80% and 65%, respectively) [29,57]. Moreover, whereas co-affinity purification gives validation of the physical interaction of proteins, the genetic modification screen provides additional information suggesting a biological role in genetic pathways relevant to HD. Overall, these observations demonstrate the utility of combining protein-interaction screening with genetic-interaction screening to provide insight into disease mechanisms and identify potential targets for therapeutic intervention.

Whereas our datasets more than quadruple the potential number of interactions attributed to Htt or Htt fragments, the in vitro derived interactor datasets do contain nonrelevant interactions (false positives) and do not represent all binding proteins (false negatives), an issue common to high-throughput screens. For example, despite the saturation of the screens we identified some, but not all, of the known Htt-fragment-interacting proteins. Our protein interaction screens revealed 14 of the 40 interactions previously discovered using Y2H methods [22,23]. Using different Y2H methods, a recent high-throughput screen isolated 19 Htt-fragment-interacting proteins, four of which had been previously described [57]. Together, these data suggest that different Y2H methods yield overlapping but not identical datasets, likely due to differences in selection stringency as well as other technical differences. Surprisingly, only Htt fragments near the N terminus of the protein were able to generate reproducible protein interaction in our Y2H screens (Table 2). This finding is consistent with a previous report in which Y2H methods failed to detect interactions from Htt-fragment baits outside the amino terminus [58] and may be in part due to technical limitations of the Y2H method. For example, C-terminal Htt fragments may not fold properly in yeast, may require post-translational modifications not found in yeast for interaction with protein partners, or may be localized away from the nucleus. Even fewer known Htt-interacting proteins were found by pull-down/MS methods. Interestingly, the cytosolic chaperonin-containing *t*-complex (CCT or TriC) was recently shown to physically interact with Htt and modify the course of polyQ-induced toxicity in mammalian cells [59,60]. We found that two components of the CCT complex, CCT6 and CCT8, were associated with Htt exon1 in pull downs. Together, these data suggest that many potential Htt-interacting or Htt-associated proteins remain to be discovered by other methods.

Overall, there was little overlap between interactions found by the Y2H and pull-down methods (4/234). This low degree of overlap is consistent with results seen in other systems-scale protein interaction datasets generated using Y2H and MS methods. For example, interaction screens of the yeast proteome using Y2H (4,476 and 915 binary protein interactions) [27,28] and MS-based screens (3,767 and 3,727 interactions in proteins complexes) [25,26] yielded a 2%–5% overlap. It has been suggested that this low overlap between interaction screening methods may arise from several factors including method-specific biases [34]. Ultimately, the value of protein interaction data generated by any method needs to be evaluated through experimental validation. We clearly demonstrate here that both methods are similarly capable of identifying Htt-fragment-interacting proteins that can be validated by assays based upon genetic interaction and physical association in mammalian tissues relevant to HD pathology.

Most specific molecular mechanisms proposed for Htt-mediated pathogenesis can, in principle, be attributed to a direct interaction between Htt and a protein component (or components) of a given pathway. Consistent with this assertion, we demonstrate here that a large set of Htt-interacting proteins is highly enriched for genetic modifiers of Htt-mediated neurodegeneration. Currently, there are efforts directed toward discovering genetic modifiers of human HD. Since the modifiers reported here were first discovered in screens performed with mammalian genes and proteins and subsequently validated in *Drosophila*, it would be of interest to determine whether human gene variants encoding similar proteins and pathway act can act as modifiers in human neurodegeneration.

## Materials and Methods

### Y2H screens.

Automated screens were done as described in LaCount et al. [31]. Briefly, haploid yeast expressing Htt-bait fusion proteins were grown in liquid medium in 96-well plates. Aliquots of yeast of the opposite mating type expressing prey libraries were added to each well and allowed to mate overnight. Matings were plated on medium selecting for diploids, the expression of the auxotrophic markers fused to the cDNA inserts and to the activity of the metabolic reporter genes *ADE2* and *HIS3* [32,61]. cDNA prey inserts from yeast that grew under selection were PCR-amplified and sequenced. Identities of prey inserts were determined by BLAST comparison against the National Center for Biotechnology (NCBI) RefSeq database (http://www.ncbi.nlm.nih.gov). All reported interactions were verified by recovering prey plasmids from positive colonies, transforming these into yeast strains expressing Htt baits and reconfirming the ADE+, HIS+ phenotype. Beta-galactosidase measurements were performed according to manufacturer's directions (Pierce, http://www.piercenet.com). Control yeast strains carrying Htt bait and prey plasmids without an insert were used as baseline. The Htt 55 Q bait had slightly higher background levels than the corresponding Htt 23 Q bait. Y2H interactor lists were filtered to remove promiscuous proteins. Additional yeast methods can be found in Supporting Information.

### MS.

Htt-fragment-interacting proteins underwent TAP and were identified by MS [62]. Affinity-tagged Htt N-terminal fragments fused to GST and 6 × His were incubated with protein lysates prepared from mouse and human tissues and cultured cells. After TAP, proteins were digested with trypsin, desalted, and subjected to strong cation exchange (CEX). CEX fractions were further separated by reverse-phase HPLC and subjected to MS analysis by matrix-assisted laser desorption/ionisation-time of flight (MALDI)MS/MS and electrospray ionization MS/MS. MS/MS data were used for protein sequence database searches by Mascot (Matrix Sciences, http://www.matrixscience.com) [63,64]. All searches were performed against the subset of either human or mouse proteins in the NCBInr protein sequence database (HumanNR or MouseNR). The minimum peptide score was set at 10, and the minimum peptide length was set to 5; otherwise the default instrument-specific Mascot settings were used. A variable cut-off was applied to proteins, which was dependent upon the number of peptides identified for a given protein. For any protein from which only one peptide was identified, a minimal peptide score threshold of 60 was required. If two peptides were identified, a threshold ion score of 50 was required, and for three peptides an ion score of 40 was required. Any peptides observed in control pull downs done with beads bound to TAP-tag alone were excluded. A statistical method, based on comparison of a wide variety of pull downs, was used to identify nonspecific interactors, which were also excluded. To validate protein identification subsequent to the automated thresholding and initial filtering, each remaining MS/MS spectrum was manually inspected to ensure that there were no spurious results matched by Mascot. Detailed MS and statistical methods can be found online with Supporting Information.

### *Drosophila* polyQ toxicity model and genetic screen.

A *Drosophila* polyQ toxicity model was generated using an N-terminal fragment of the human *HD* cDNA that encodes the first 336 amino acids of the protein including a 128-Q expansion in exon 1. The construct was cloned into the pUAST vector for generating transgenic lines [65]. This HD *Drosophila* model is most similar to the expanded version (82 Q) of the N171 mouse model, which shows abundant intranuclear inclusions [66] and neuronal degeneration [67]. Expression of the 128-Q N-terminal Htt fragment in *Drosophila* leads to neurodegenerative phenotypes. In the eye, these phenotypes are evident both externally and in the retina following expression using the glass multimer reporter (*GMR)-*GAL4 driver (Figures 2 and 3). In the nervous system, *Elav-*GAL4-directed expression of the transgene leads to progressive impaired motor ability and reduced life span (Figure 3C). Also as in the N171-82Q mouse, intranuclear inclusions are observed in *Drosophila* neurons expressing the 128-Q N-terminal Htt fragment (unpublished data).

For the modifier screen, females of the genotype *y^1^w^118^; GMR-GAL4/CyO; UAS:128QHtt[M64]* were crossed to males from the mutant strains. In cases where the mutation was on the X chromosome, the cross was reversed. Crosses were incubated at 27 °C and 29 °C to provide two different phenotypic readouts. Strains modifying the eye phenotype were recrossed to verify the modification. Only genes that consistently showed modification at different temperatures or using different alleles were further analyzed. Potential modifiers behaving as enhancers were tested for possible nonspecific eye phenotypes by crossing them to control females of the genotype *y^1^w^118^; GMR-GAL4/CyO.*

For scanning electron microscopy (SEM) images, flies were crossed at 29 °C and newly eclosed adults were aged for five days. Whole flies were dehydrated in ethanol, critical-point dried, and analyzed with a JEOL JSM 6100 microscope. For paraffin sections of enhancers, flies were crossed at 25 °C and adults were aged for five days (for suppressors, the crosses were done at 27 °C and the flies were aged for one day). Adult heads and torsos were fixed in 4% formaldehyde/85% ethanol/5% acetic acid, dehydrated, embedded in paraffin for vertical semi-thin sections, and then stained with Hemathox.

For the climbing and survival assays, females of the genotype Elav-GAL4; UAS:128QHtt[F27B] were crossed to males of the mutant strains. Climbing assays were performed on 30 age-matched adult virgin female flies raised at 27 °C as described [68]. The flies, placed in a plastic vial, were tapped to the bottom of the vial, and the number of flies above a 5-cm line was counted after 18 seconds. A total of ten trials were performed every 48 hours. Each climbing and survival experiment was repeated three times.

### Immunoprecipitation.

Whole brains from wild-type or YAC128 mice were lysed in T-PER (Pierce) with protease inhibitors (Complete Mini, Roche Applied Science, http://www.roche.com). Protein determination was carried out with the BCA method (Bio-Rad, http://www.bio-rad.com). Lysate (500 μg, 0.7 ml T-PER with protease inhibitors) were precleared with mouse IgG beads (Sigma A6531, http://www.sigmaaldrich.com) and immunoprecipitated with monoclonal Htt antibody (5 μl, Chemicon 2166, http://www.chemicon.com) by incubating overnight at 4 °C and then with protein G (40 μl, Amersham 17-0618-01, http://www.amersham.com). Beads were washed 5× with TBS/0.05% Tween, sample was eluted with 1× sample buffer (Invitrogen, http://www.invitrogen.com) and then resolved using 4%–12% Bis-Tris precast gels (Invitrogen). Western blot was preformed, and blots were probed with rabbit antibody to USP9X (1:200, Abcam 19879, http://www.abcam.com), Cullin 2 (1:500, Abcam 1870), CACNA2D1 (1:200, Sigma CS105), Htt BKP1 (1:500), PARP (1:300, BioMol SA253, http://www.biomol.com), mouse monoclonal GAPDH (1:100, Chemicon MAB374), STX1A (1:1000, Synaptic Systems, 11001, http://www.sysy.com), SNAP25 (1:1000, Santa Cruz Biotechnology SC-7539, http://www.scbt.com/), goat antibody PKM2 (1:500, Abcam 6191), and PSMC2 (1:1000, GeneTex 23322, http://www.genetex.com).

## Supporting Information

Figure S1 Purified Htt Exon 1 Bait Purified bait protein from the first and second purification steps was separated by sodium dodecyl sulfate polyacrylamide gel electrophoresis (SDS-PAGE) and silver stained. The presence of glutathione S-transferase (GST) and Htt in the bands was confirmed by matrix-assisted laser desorption/ionisation-time of flight MS and Western blotting (not shown). The predicted size of the GST-Htt fusion product is 53 kDa. We were unable to determine the difference in the two GST-Htt bands by MS; they may represent expanded (48 Q) and wild-type (22 Q) Htt fragments. The band at 28 kDa represents GST and likely occurs from cleavage of the fusion product between the GST and the bait as we saw a band of this size with numerous heterologous purified baits.
(575 KB PDF)

Figure S2 Saturation of Y2H Searches with Htt Baits Only searches with N-terminal baits (amino acid 1–90, 23 Q; amino acid 1–90, 55 Q; amino acid 1–450, 23Q; amino acid 1–450, 55 Q) that gave at least one positive were included in the analysis. The x-axis indicates numbers of screens performed. The y-axis shows the novel discovery index for prey proteins (e.g., a value of 0.3 indicates that 30% of the preys seen in a search were not seen in a prior screen). A peak near 525 searches corresponds to introduction of new prey libraries.
(529 KB PDF)

Figure S3 Suppressors of Fly Eye Phenotype Retinal sections of day-1 control flies cultured at 27 °C expressing the gene that encodes expanded N-terminal Htt *(GMR-GAL4/+; UAS:128Qhtt[M64]/+)* (B) show a severe degenerative phenotype when compared to (A) *GMR-GAL4* controls of the same age and cultured at the same temperature. The phenotype consists of a shortening (see arrow) and detachment of the retina, as well as the presence of vacuoles in the retina. The Htt-induced phenotype can be suppressed by (C) reduced levels of armadillo *(P{lacW}arm^G0234^; GMR-GAL4/+; UAS:128Qhtt[M64*]/*+),* (D) reduced levels of hu li tai shao *(GMR-GAL4/P{lacW}hts^k06121^; UAS:128Qhtt[M64]/+),* (E) reduced levels of M6 *(GMR-GAL4/+; UAS:128Qhtt[M64]/P{GT1}M6^BG00390^),* (F) reduced levels of kinesin heavy chain *(GMR-GAL4/b^1^pr^1^Khc^8^;* UAS:128Qhtt[M64*]/+),* (G) reduced levels of peanut *(GMR-GAL4/P{SUP pr P-}pnut^KG00478^; UAS:128Qhtt[M64]/+),* (H) reduced levels of 14-3-3e *(GMR-GAL4/+; UAS:htt[M64]/14-3-3e^j2b10^),* (I) reduced levels of G protein I a-subunit 65A *(GMR-GAL4/; UAS:128Qhtt[M64]/P{SUPor-P}G-ia65A^KG01907^ry^506^),* (J) reduced levels of Itp-r83A *(GMR-GAL4/+; UAS:128Qhtt[M64]/P{PZ}Itp-r83A^05616^ry^506^),* (K) over-expression of Src oncogene at 42A *(GMR- GAL4/P{EPgy2}Src42A^EY08937^; UAS:128Qhtt[M64]/+),* (L) reduced levels of clathrin heavy chain *(Chc^4^/+ GMR-GAL4/+; UAS:128Qhtt[M64]/+),* (M) reduced levels of soluble N-ethylmaleimide-sensitive factor attachment protein *(GMR-GAL4/+; UAS:128Qhtt[M64]/SNAP^M4^),* (N) reduced levels of STX1A *(GMR-GAL4/+; UAS:128Qhtt[M64]/ry^506^P{PZ}Syx1A^06737^),* (O) reduced levels of Rpt1 *(GMR-GAL4/P{PZ}Rpt1^05643cn1^; UAS:128Qhtt[M64]/+),* (P) reduced levels of Eip75B *(GMR-GAL4/+; UAS:128Qhtt[M64]/P{PZ}Eip75B^07041^),* (Q) reduced levels of myocyte enhancing factor 2 *(GMR-GAL4/Df(2R)X1,Mef2^X1^; UAS:128Qhtt[M64]/+),* (R) reduced levels of crooked legs *(GMR*-GAL4/P{EPgy2}crol^EY08953^ *),* (S) reduced levels of Glycerol 3 phosphate dehydrogenase *(GMR-GAL4/Al^1^Gpdh^n1–4^; UAS:128Qhtt[M64]/+),* (T) reduced levels of Pdsw *(GMR-GAL4/P{PlacZ}Pdsw^k10101^; UAS:128Qhtt[M64]/+),* and (U) reduced levels of porin *(GMR-GAL4/p{PlacW}porin^k05123^; UAS:128Qhtt[M64]/+)* and reduced levels of CG12455 *(GMR-GAL4/P{SUP or-P}CG12455^KG00260^; UAS:128Qhtt[M64]/+).* These mutations decrease the vacuolization and increase the retinal thickness as well as virtually eliminating the retinal detachment.
(2.7 MB PDF)

Figure S4 Enhancers of Fly Eye Phenotype (A) Age-matched controls cultured at the same temperature *(GMR-GAL4*/*+)*.
(B) Retinal sections of day 5 flies expressing N-terminal 128-Q htt *(GMR-GAL4*/+; *UAS:128Qhtt[M64]*/*+)* cultured at 25 °C show a degenerative phenotype. The phenotype consists of a shortening (arrow), vacuolization, and detachment of the retina. This phenotype can be enhanced by (C) reduced levels of *CAP (GMR-GAL4/P{SUPor-P}CAP^KG00083^; UAS:128Qhtt[M64]/+),* (D) reduced levels of *CLIP-190 (GMR-GAL4/P{SUPor-P}CLIP-190^KG06490^; UAS:128Qhtt[M64]/+),* (E) reduced levels of *LaminC (GMR-GAL4/P{PTTor-GB}LamC^G00158^; UAS:128Qhtt[M64]/+),* (F) overexpression of *M6 (GMR-GAL4/+; UAS:128Qhtt[M64]/P{EPgy2}M6^EY07032^),* (G) reduced levels of *zipper (GMR-GAL4/P{PZ}zip^02957^; UAS:128Qhtt[M64]/+),* (H) reduced levels of *short stop (GMR-GAL4/P{FRT(w^hs^)}G13 shot^3^; UAS:128Qhtt[M64]/+),* (I) overexpression of *14-3-3ɛ (GMR-GAL4/+; UAS:128Qhtt[M64]/14-3-3ɛ^ Scer\UASc.Ca^),* (J) overexpression of *14-3-3ς (GMR-GAL4/ P{EPgy2}14-3-3ς^EY03325^; UAS:128Qhtt[M64]/+),* (K) overexpression of *G-iα65A (GMR-GAL4/ +; UAS:128Qhtt[M64]/P{EPgy2}G-iα65A^EY10355^),* (L) overexpression of *Itp-r83A (GMR-GAL4/ +; UAS:128Qhtt[M64]/P{EPgy2}Itp-r83A^EY02522^),* (M) reduced levels of *Lachesin (GMR-GAL4/P{PTT-un1}Lac^G00044^*; *UAS:128Qhtt[M64]*/*+),* (N) reduced levels of *Src oncogene at 42A (GMR-GAL4/P{lacW}Src42A^k10108^*; *UAS:128Qhtt[M64]*/*+),* (O) overexpression of *soluble NSF-attachment protein (GMR-GAL4*/+; *UAS:128Qhtt[M64]*/*UAS-S102C#2D),* (P) overexpression of *Syntaxin1A* (*GMR-GAL4*/+; *UAS:128Qhtt[M64]*/*P{EP}Syx1A^EP3215^),* (Q) reduced levels of *Aspartyl β-hydroxylase (GMR-GAL4/P{SUPor-P}Asph^KG09881^*; *UAS:128Qhtt[M64]*/*+),* (R) reduced levels of *Dynein heavy chain 64C (GMR-GAL4*/+; *UAS:128Qhtt[M64]*/*P{SUPor-P}Dhc64C^KG08838^),* (S) reduced levels of *fat facets (GMR-GAL4*/+; *UAS:128Qhtt[M64]*/*faf^Bx4^),* (T) overexpression of *Rpt1 (GMR-GAL4/P{EP}Rpt1^EP2153^*; *UAS:128Qhtt[M64]*/*+),* (U) reduced levels of *crooked legs (GMR-GAL4/P{PZ}crol^04418^; UAS:128Qhtt[M64]*/*+),* (V) reduced levels of *Phosphogluconate isomerase (GMR-GAL4/Pgi^nNC1^; UAS:128Qhtt[M64]*/*+),* (W) reduced levels of *RhoGAP92B (GMR-GAL4/P{UAS-RhoGAP92B-dsRNA}2.2; UAS:128Qhtt[M64]*/*+),* (X) reduced levels of *Unc-76 (P{lacW}Unc-76^G0423a^; GMR-GAL4/+; UAS:128Qhtt[M64]*/*+),* and (Y) overexpression of *CG12455 (GMR-GAL4/P{EPgy2}CG12455^EY09750^; UAS:128Qhtt[M64]*/*+)*. These mutations do not cause an abnormal eye phenotype in control flies carrying the *GMR-GAL4* driver without the *UAS:128Qhtt[M64]* transgene (unpublished data). However, when combined with 128-Q htt, they lead to further decrease in retinal thickness and in some cases increased retinal detachment and vacuolization.
(2.8 MB PDF)

Table S1 Primary List of Peptides Identified in Pull Downs Y indicates peptides that were manually validated and confirmed by inspection of the MS spectra; A refers to ambiguous peptides that could not be conclusively identified by manual validation of the MS spectra.
(3.4 MB XLS)

Table S2 Primary List of Gene Sequences Identified from Y2H Positives with Htt-Fragment Baits *The total number of unique interacting proteins refers to the number of unique gene sequences identified in a database of positives from nearly >250,000 high-throughput random Y2H searches performed at Prolexys Pharmaceuticals (http://www.prolexys.com).
(580 KB DOC)

Table S3 Sequences of Positives Identified in Y2H Searches Search ID is an identifier given each Y2H mating event (see Materials and Methods). Positive ID is a unique identifier given to each positive colony picked in Y2H searches. RefSeq ID, Gene Symbol, and Entrez Gene ID refer to gene designations in the NCBI database (http://www.ncbi.nlm.nih.gov/entrez/query.fcgi?db=gene). E_VALUE_EXP is the negative log of the E value produced by the highest scoring BLAST hit and has a maximum of 180 (corresponds to E value of 10E^−180^ or less). Amino acid coordinates of HD baits are indicated relative to NP_002102. Q-length repeats are shown in parentheses. HD bait sequences may be represented multiple times if more than one search generated positives. High-throughput sequencing was performed unidirectionally for identification purposes and does not necessarily represent the entirety of the clone. *Search ID 14291 (HD bait 1116–1196) was identified in a search using a complex bait library rather than individual bait clone.
(3.2 MB XLS)

Table S4 *Drosophila* Orthologs of Human Genes Tested in the Fly HD Strain (98 KB DOC)

Table S5 *Drosophila* Modifiers with Only One Confirmed Modification Result *Genes tested in *Drosophila* prior to statistical filtering; E, Enhancer; S, Suppressor
(73 KB DOC)

### Accession Numbers

The National Center for Biotechnology Information (NCBI) (http://www.ncbi.nlm.nih.gov/entrez/query.fcgi?db=Protein) accession numbers for MS studies (RefSeq) are: NP_000302.1, NP_000382.3, NP_000524.3, NP_000602.1, NP_000708.1, NP_001019645, NP_001367.2, NP_001377.1, NP_001419.1, NP_001753.1, NP_001779.2, NP_001834.2, NP_001853.2, NP_001854.1, NP_001907.2, NP_001914.2, NP_001951.2, NP_001990.1, NP_002046.1, NP_002064.1, NP_002065.1, NP_002102.4, NP_002329.2, NP_002536.1, NP_003033.2, NP_003124.1, NP_003156.1, NP_003170.1, NP_003356.2, NP_003357.2, NP_003365.1, NP_003366.2, NP_003696.2, NP_004246.1, NP_004309.2, NP_004364.2, NP_004365.1, NP_004484.1, NP_004491.1, NP_004539.1, NP_004542.1, NP_004543.1, NP_004594.1, NP_004850.1, NP_004993.1, NP_004996.1, NP_004997.4, NP_005156.1, NP_005264.2, NP_005268.1, NP_005653.3, NP_005736.3, NP_005995.1, NP_006046.1, NP_006279.2, NP_006308.3, NP_006576.2, NP_006810.1, NP_006830.1, NP_008839.2, NP_009034.2, NP_009204.1, NP_031407.2, NP_031457.1, NP_031464.1, NP_031669.2, NP_031736.1, NP_031773.1, NP_031887.2, NP_031959.1, NP_032246.2, NP_032518.1, NP_032644.2, NP_033012.1, NP_033033.1, NP_033321.1, NP_033332.1, NP_033333.2, NP_033441.1, NP_033805.1, NP_033851.1, NP_033914.1, NP_034053.1, NP_034078.1, NP_034438.1, NP_034442.1, NP_034715.1, NP_034829.1, NP_034944.1, NP_035229.2, NP_035253.1, NP_035523.1, NP_035558.1, NP_035824.1, NP_035825.1, NP_036288.2, NP_036560.1, NP_036611.2, NP_038709.1, NP_057049.3, NP_057223.1, NP_057606.1, NP_058084.2, NP_060064.2, NP_061359.2, NP_062681.1, NP_065593.1, NP_066268.1, NP_067541.1, NP_075553.1, NP_077128.2, NP_077173.1, NP_077725.1, NP_077745.2, NP_079589.1, NP_079612.1, NP_079634.1, NP_079683.2, NP_080175.1, NP_080720.1, NP_080971.2, NP_080979.1, NP_084501.1, NP_114080.2, NP_149124.2, NP_443106.1, NP_444427.1, NP_536846.1, NP_536849.1, NP_542970.1, NP_570824.1, NP_598429.1, NP_613063.1, NP_619621.1, NP_659409.2, NP_663493.1, NP_663589.2, NP_766024.1, NP_776169.2, NP_796376.2, NP_849209.1, NP_976218.1, XP_128725.4, XP_131103.3, XP_203393.2, and XP_622887.1.

The NCBI (GeneID) (http://www.ncbi.nlm.nih.gov/entrez/query.fcgi?db=gene) accession numbers for Y2H studies are: 120, 161, 323, 1315, 1387, 1499, 1759, 1778, 1785, 2597, 3064, 3092, 3093, 3275, 3329, 3338, 3839, 4209, 4361, 4790, 5033, 5295, 5296, 5315, 5468, 5493, 5710, 5753, 6670, 6721, 6829, 6867, 7430, 7529, 7644, 7692, 7704, 7802, 8065, 8239, 8453, 8462, 8503, 8539, 9093, 9330, 9611, 9638, 9810, 9818, 9901, 9938, 10010, 10133, 10422, 10456, 10458, 10464, 10540, 10580, 10906, 10915, 11177, 11193, 23116, 23328, 23332, 23348, 23360, 23380, 23609, 23613, 23641, 25764, 26578, 27068, 28969, 29062, 29072, 29993, 51061, 51322, 51586, 51593, 51667, 55219, 55660, 55704, 55735, 56254, 57489, 57509, 57522, 57616, 63908, 79027, 79813, 80254, 83478, 84936, 128866, 134218, 139818, 152789, and 171392.

## Abbreviations

CCT: chaperonin-containing *t-complex* polypeptide 1
HD: Huntington's disease
Htt: huntingtin
MS: mass spectrometry
polyQ: polyglutamine
Q: glutamine
TAP: tandem affinity purification
Y2H: yeast two hybrid
