# Supplementary material for: Huntingtin Interacting Proteins Are Genetic Modifiers of Neurodegeneration
Source: PLoS Genet. 2007 May 11;3(5):e82. doi: 10.1371/journal.pgen.0030082 (PMC1866352; doi:10.1371/journal.pgen.0030082)
Supplement: Supplementary file 1 [file pgen.0030082.sg001.pdf]

Figure S1

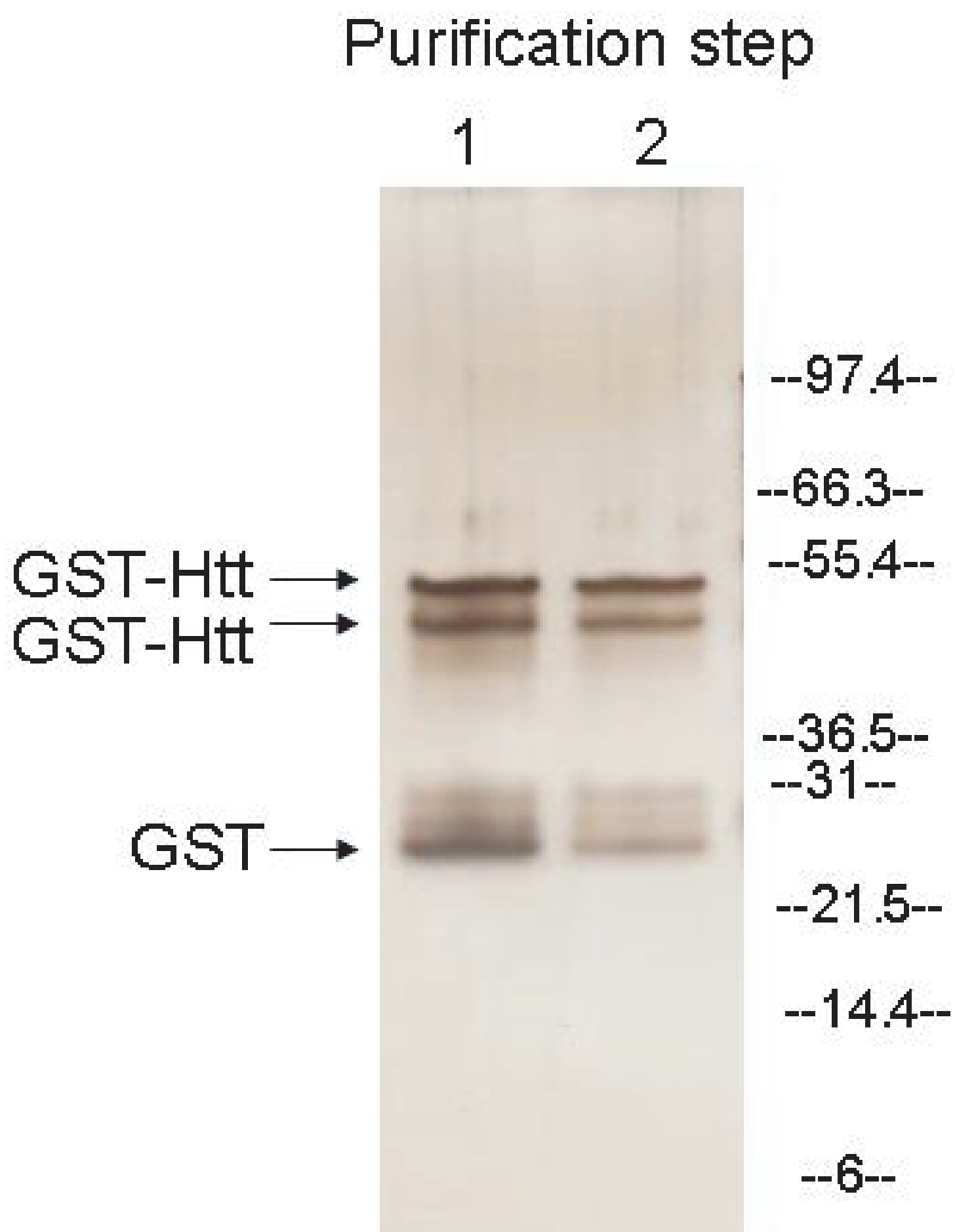
Purification step
1
2
--97.4--
--66.3--
--55.4--
GST-Htt
GST-Htt
--36.5--
--31--
GST
--21.5--
--14.4--
--6--
