# Supplementary material for: Huntingtin Interacting Proteins Are Genetic Modifiers of Neurodegeneration
Source: PLoS Genet. 2007 May 11;3(5):e82. doi: 10.1371/journal.pgen.0030082 (PMC1866352; doi:10.1371/journal.pgen.0030082)
Supplement: Supplementary file 2 [file pgen.0030082.sg002.pdf]

Figure S2

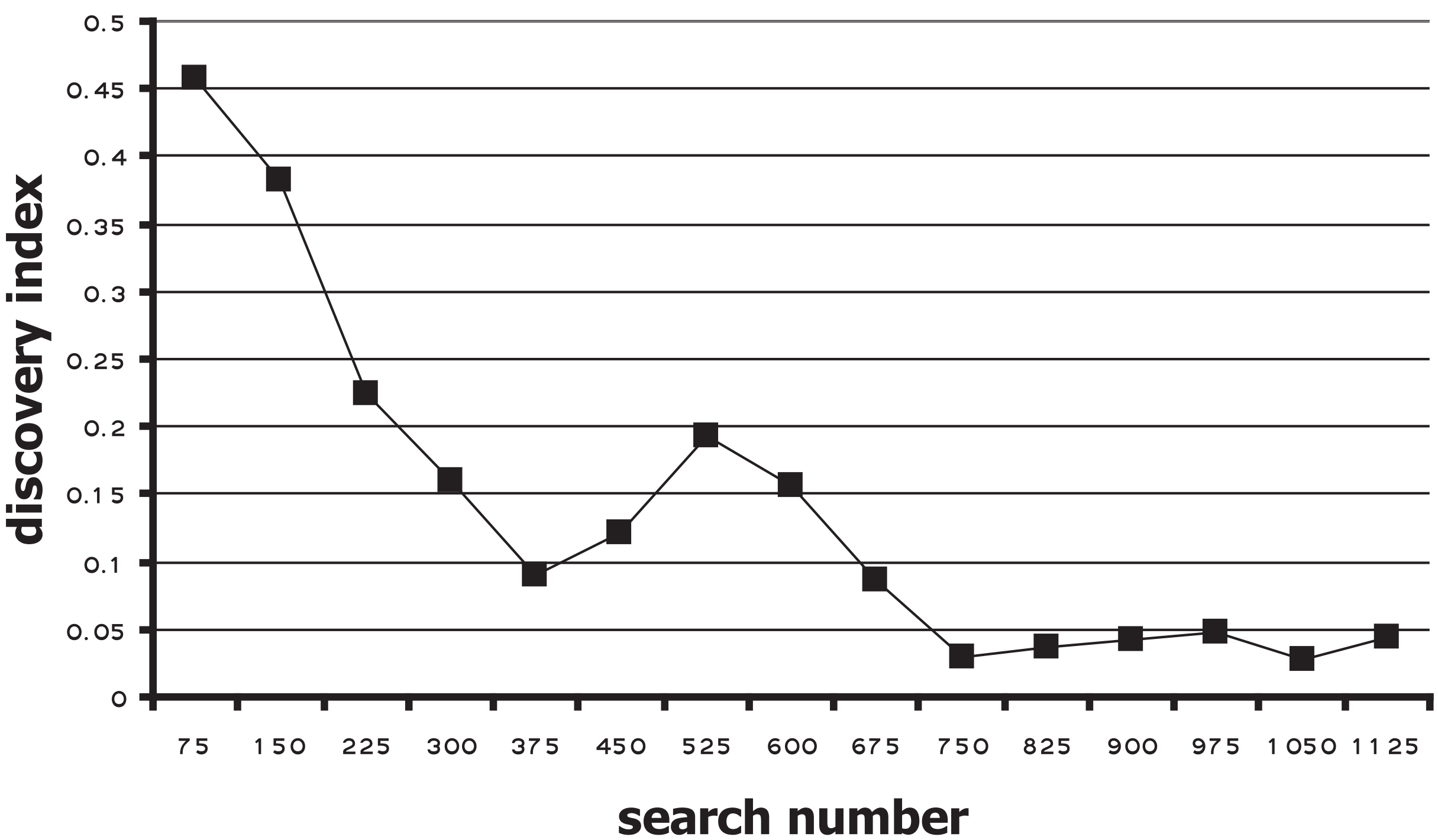

0.5
0.45
0.4
0.35
0.3
0.25
0.2
0.15
0.1
0.05
0
discovery index
75
150
225
300
375
450
525
600
675
750
825
900
975
1050
1125
search number
