# Supplementary material for: Huntingtin Interacting Proteins Are Genetic Modifiers of Neurodegeneration
Source: PLoS Genet. 2007 May 11;3(5):e82. doi: 10.1371/journal.pgen.0030082 (PMC1866352; doi:10.1371/journal.pgen.0030082)

Figure S3

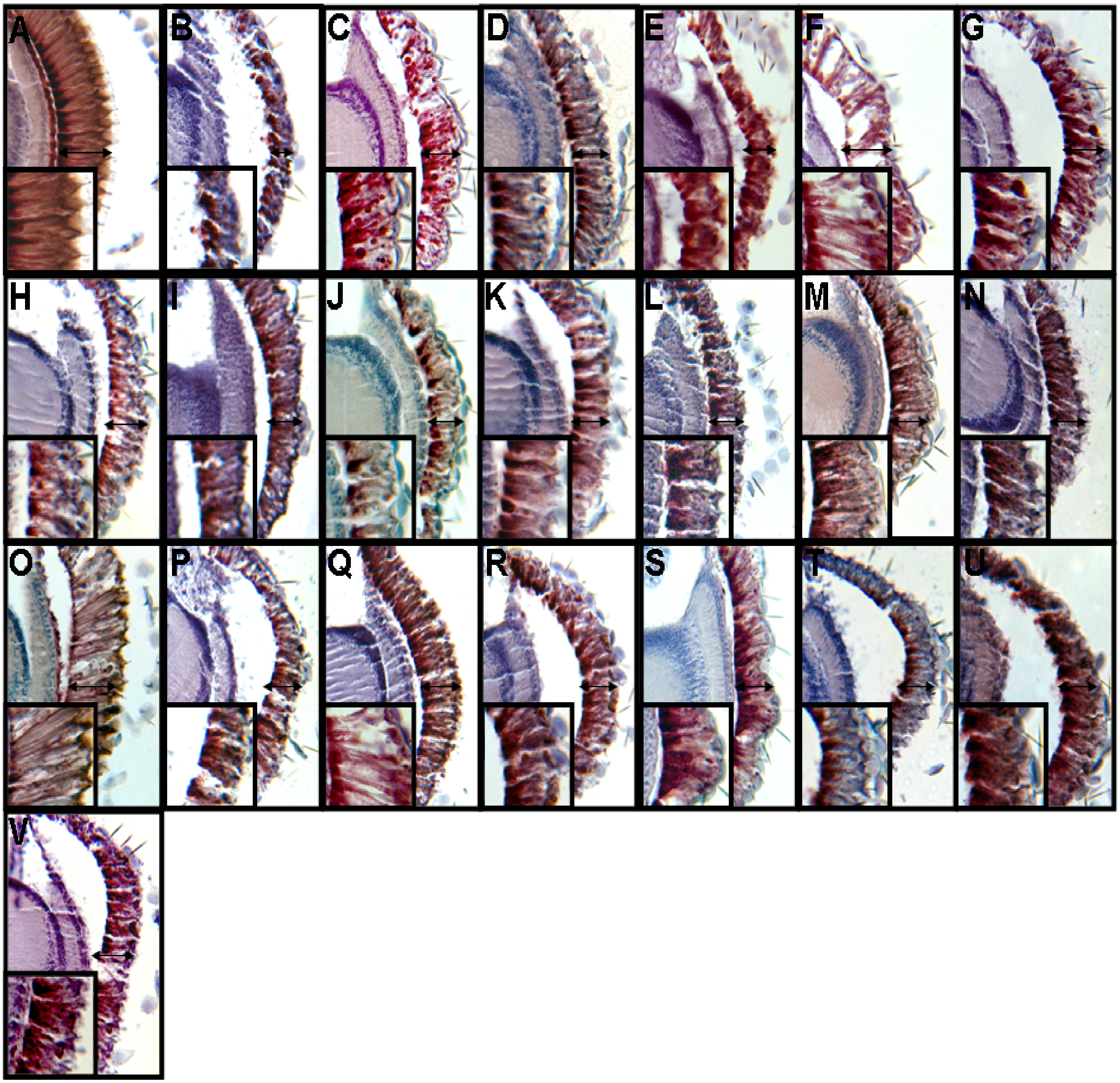

Supplement: Supplementary file 3 [file pgen.0030082.sg003.pdf]
