# Supplementary material for: Huntingtin Interacting Proteins Are Genetic Modifiers of Neurodegeneration
Source: PLoS Genet. 2007 May 11;3(5):e82. doi: 10.1371/journal.pgen.0030082 (PMC1866352; doi:10.1371/journal.pgen.0030082)
Supplement: Supplementary file 5 [file pgen.0030082.st002.doc]

| locus symbol | locus ID | number of unique interacting proteins* | total number of Htt positives |
| --- | --- | --- | --- |
| 384D8-2 | 29781 | 10 | 2 |
| AATF | 26574 | 37 | 2 |
| ABLIM3 | 22885 | 25 | 1 |
| ABR | 29 | 62 | 1 |
| ACTN1 | 87 | 320 | 20 |
| ACTN4 | 81 | 506 | 8 |
| ADD3 | 120 | 42 | 3 |
| ADH5 | 128 | 33 | 1 |
| AF5Q31 | 27125 | 23 | 1 |
| AKAP9 | 10142 | 267 | 1 |
| AKNA | 80709 | 19 | 1 |
| AKT3 | 10000 | 24 | 1 |
| ALB | 213 | 187 | 1 |
| ALDH3A2 | 224 | 93 | 1 |
| AMY2B | 280 | 2 | 1 |
| ANK3 | 288 | 154 | 1 |
| ANKFY1 | 51479 | 18 | 1 |
| ANKRD11 | 29123 | 130 | 1 |
| ANKRD12 | 23253 | 73 | 1 |
| ANKRD26 | 22852 | 59 | 8 |
| AP1B1 | 162 | 45 | 1 |
| AP2A2 | 161 | 17 | 2 |
| AP2B1 | 163 | 137 | 1 |
| AP3D1 | 8943 | 78 | 2 |
| APBB2 | 323 | 31 | 4 |
| APG3 | 64422 | 11 | 1 |
| API5 | 8539 | 46 | 3 |
| APLP2 | 334 | 305 | 7 |
| APOL1 | 8542 | 15 | 5 |
| APP | 351 | 340 | 1 |
| APRIN | 23047 | 42 | 1 |
| ARFIP1 | 27236 | 38 | 1 |
| ARHGAP22 | 58504 | 20 | 3 |
| ARHGAP24 | 83478 | 9 | 18 |
| ARHGAP25 | 9938 | 6 | 3 |
| ARHGAP4 | 393 | 10 | 1 |
| ARHGAP5 | 394 | 21 | 1 |
| ARNT | 405 | 20 | 1 |
| ARRB1 | 408 | 579 | 1 |
| ARS2 | 51593 | 68 | 19 |
| ATP1A3 | 478 | 79 | 1 |
| ATP2B1 | 490 | 41 | 1 |
| ATP5L | 10632 | 3 | 4 |
| ATRX | 546 | 116 | 1 |
| AXOT | 64844 | 48 | 1 |
| BAI2 | 576 | 90 | 1 |
| BAIAP2 | 10458 | 249 | 14 |
| BAT2 | 7916 | 67 | 1 |
| BAZ1A | 11177 | 50 | 9 |
| BCAS1 | 8537 | 92 | 1 |
| BDP1 | 55814 | 86 | 2 |
| BEXL1 | 56271 | 26 | 1 |
| BHLHB3 | 79365 | 51 | 2 |
| BLM | 641 | 4 | 1 |
| BMP2K | 55589 | 14 | 2 |
| BPAG1 | 667 | 971 | 310 |
| BRD7 | 29117 | 50 | 1 |
| BZW2 | 28969 | 15 | 2 |
| C12ORF2 | 11228 | 30 | 1 |
| C13ORF24 | 10464 | 67 | 8 |
| C14ORF32 | 93487 | 11 | 1 |
| C20ORF178 | 128866 | 63 | 22 |
| C20ORF6 | 51575 | 41 | 1 |
| C21ORF70 | 85395 | 11 | 4 |
| C6ORF111 | 25957 | 208 | 1 |
| C6ORF93 | 84946 | 33 | 2 |
| CALM2 | 805 | 65 | 1 |
| CASKIN1 | 57524 | 27 | 2 |
| CCAR1 | 55749 | 46 | 2 |
| CCNT2 | 905 | 14 | 1 |
| CD22 | 933 | 15 | 1 |
| CDC40 | 51362 | 10 | 2 |
| CDGAP | 57514 | 9 | 1 |
| CDR1 | 1038 | 107 | 1 |
| CDS2 | 8760 | 10 | 3 |
| CENPJ | 55835 | 96 | 1 |
| CEP2 | 11190 | 176 | 2 |
| CHAF1A | 10036 | 38 | 1 |
| CHD4 | 1108 | 115 | 1 |
| CHGB | 1114 | 112 | 2 |
| CKAP4 | 10970 | 28 | 1 |
| CKM | 1158 | 4 | 1 |
| CLASP1 | 23332 | 43 | 4 |
| CLTC | 1213 | 432 | 6 |
| CLU | 1191 | 206 | 2 |
| CNN3 | 1266 | 44 | 1 |
| CNOT2 | 4848 | 62 | 5 |
| COG6 | 57511 | 14 | 1 |
| COL1A2 | 1278 | 82 | 3 |
| COL4A3BP | 10087 | 15 | 2 |
| COPB | 1315 | 60 | 3 |
| COPS5 | 10987 | 2477 | 6 |
| CPA1 | 1357 | 15 | 1 |
| CPB1 | 1360 | 45 | 3 |
| CREBBP | 1387 | 569 | 1 |
| CRYAB | 1410 | 76 | 1 |
| CSN2 | 1447 | 479 | 13 |
| CSNK1D | 1453 | 7 | 1 |
| CSNK2B | 1460 | 220 | 1 |
| CTNNB1 | 1499 | 1431 | 11 |
| CUL2 | 8453 | 16 | 3 |
| CUL3 | 8452 | 58 | 1 |
| CUL5 | 8065 | 14 | 6 |
| CUL7 | 9820 | 12 | 1 |
| CX3CR1 | 1524 | 5 | 1 |
| D21S2056E | 8568 | 25 | 1 |
| DBC-1 | 57805 | 15 | 1 |
| DCTN2 | 10540 | 90 | 59 |
| DDX17 | 10521 | 63 | 1 |
| DHX9 | 1660 | 53 | 1 |
| DIP13B | 55198 | 36 | 2 |
| DKFZP434G156 | 64753 | 127 | 1 |
| DKFZP586M1819 | 137964 | 6 | 1 |
| DNAJA1 | 3301 | 39 | 1 |
| DNAJA3 | 9093 | 19 | 14 |
| DNAJA5 | 134218 | 90 | 23 |
| DNAJC11 | 55735 | 15 | 11 |
| DNAJC4 | 3338 | 9 | 20 |
| DNALI1 | 7802 | 10 | 1 |
| DNCH1 | 1778 | 241 | 6 |
| DNM1 | 1759 | 185 | 4 |
| DNM2 | 1785 | 30 | 3 |
| DOCK11 | 139818 | 11 | 4 |
| DOCK9 | 23348 | 45 | 10 |
| DRG1 | 4733 | 4 | 1 |
| DRPLA | 1822 | 93 | 1 |
| DXS9928E | 9130 | 36 | 1 |
| EEA1 | 8411 | 32 | 2 |
| EEF1G | 1937 | 173 | 2 |
| EHMT1 | 79813 | 1 | 3 |
| EIF2S2 | 8894 | 29 | 1 |
| EIF3S2 | 8668 | 2 | 1 |
| EIF3S9 | 8662 | 17 | 1 |
| ENO1 | 2023 | 85 | 1 |
| ERBB2 | 2064 | 35 | 1 |
| ESRRBL1 | 55081 | 239 | 1 |
| EST1B | 23381 | 20 | 1 |
| ETV1 | 2115 | 2 | 1 |
| FAF1 | 11124 | 17 | 1 |
| FALZ | 2186 | 115 | 1 |
| FBXL11 | 22992 | 456 | 1 |
| FEZ1 | 9638 | 68 | 6 |
| FKBP3 | 2287 | 10 | 1 |
| FLJ10211 | 55095 | 29 | 1 |
| FLJ10378 | 55132 | 22 | 3 |
| FLJ11457 | 79809 | 24 | 1 |
| FLJ12949 | 65095 | 37 | 2 |
| FLJ13305 | 84140 | 37 | 1 |
| FLJ13386 | 80254 | 85 | 5 |
| FLJ20086 | 54816 | 14 | 1 |
| FLJ20241 | 54862 | 27 | 1 |
| FLJ20628 | 55006 | 3 | 1 |
| FLJ20729 | 54680 | 3 | 1 |
| FLJ21174 | 79921 | 49 | 1 |
| FLJ22965 | 63932 | 23 | 1 |
| FLJ25005 | 123283 | 57 | 2 |
| FLJ25286 | 153443 | 606 | 1 |
| FLJ35827 | 256364 | 6 | 1 |
| FLJ38984 | 127703 | 24 | 1 |
| FLJ39441 | 144108 | 4 | 1 |
| FLJ39739 | 388685 | 2 | 1 |
| FLNA | 2316 | 719 | 3 |
| FLNB | 2317 | 272 | 13 |
| FMNL2 | 114793 | 122 | 1 |
| FMOD | 2331 | 10 | 1 |
| FNBP4 | 23360 | 85 | 175 |
| FNTA | 2339 | 31 | 3 |
| FTH1 | 2495 | 699 | 2 |
| FTL | 2512 | 193 | 217 |
| FUBP1 | 8880 | 82 | 1 |
| FUCA1 | 2517 | 5 | 1 |
| FUS | 2521 | 305 | 1 |
| FUS2 | 24142 | 16 | 1 |
| GA17 | 10480 | 25 | 1 |
| GAK | 2580 | 15 | 1 |
| GAPD | 2597 | 252 | 1 |
| GAS7 | 8522 | 165 | 6 |
| GCC2 | 9648 | 250 | 7 |
| GFAP | 2670 | 365 | 5 |
| GIT1 | 28964 | 56 | 1 |
| GLTSCR2 | 29997 | 351 | 1 |
| GOLGB1 | 2804 | 330 | 3 |
| GRINL1A | 81488 | 6 | 1 |
| GRIPAP1 | 56850 | 135 | 1 |
| GRP58 | 2923 | 108 | 1 |
| GSN | 2934 | 55 | 1 |
| GSTP1 | 2950 | 8 | 4 |
| GTF2I | 2969 | 185 | 1 |
| GTF3C3 | 9330 | 39 | 4 |
| HAPIP | 8997 | 316 | 9 |
| HAX1 | 10456 | 123 | 7 |
| HDAC9 | 9734 | 19 | 1 |
| HDLBP | 3069 | 196 | 2 |
| HEPH | 9843 | 1 | 1 |
| HERC1 | 8925 | 59 | 1 |
| HIP1 | 3092 | 25 | 10 |
| HIP2 | 3093 | 61 | 4 |
| HIPK2 | 28996 | 108 | 2 |
| HK1 | 3098 | 26 | 1 |
| HMG2L1 | 10042 | 14 | 1 |
| HMGB2 | 3148 | 53 | 7 |
| HMGCR | 3156 | 20 | 1 |
| HNRPA1 | 3178 | 313 | 2 |
| HNRPA2B1 | 3181 | 422 | 1 |
| HNRPH1 | 3187 | 86 | 1 |
| HNRPU | 3192 | 148 | 1 |
| HOM-TES-103 | 25900 | 11 | 1 |
| HRI | 27102 | 16 | 1 |
| HRMT1L1 | 3275 | 8 | 4 |
| HSGT1 | 11319 | 23 | 3 |
| HSPA4 | 3308 | 59 | 3 |
| HSPA5 | 3309 | 302 | 5 |
| HSPC049 | 29062 | 13 | 4 |
| HSPC148 | 51503 | 13 | 1 |
| HSPCA | 3320 | 758 | 2 |
| HSPD1 | 3329 | 91 | 1 |
| HSPG2 | 3339 | 124 | 1 |
| HSPH1 | 10808 | 89 | 1 |
| HUMPPA | 30850 | 4 | 2 |
| HYPB | 29072 | 86 | 37 |
| HYPK | 25764 | 12 | 1 |
| IQGAP2 | 10788 | 74 | 2 |
| ITSN2 | 50618 | 129 | 1 |
| KIAA0056 | 23310 | 14 | 1 |
| KIAA0372 | 9652 | 34 | 1 |
| KIAA0376 | 23384 | 183 | 1 |
| KIAA0406 | 9675 | 7 | 1 |
| KIAA0423 | 23116 | 19 | 10 |
| KIAA0555 | 9832 | 28 | 1 |
| KIAA0599 | 26030 | 28 | 4 |
| KIAA0853 | 23091 | 98 | 1 |
| KIAA1202 | 57477 | 20 | 1 |
| KIAA1212 | 55704 | 2 | 3 |
| KIAA1229 | 57489 | 27 | 4 |
| KIAA1244 | 57221 | 11 | 1 |
| KIAA1361 | 57551 | 34 | 2 |
| KIAA1387 | 57223 | 31 | 1 |
| KIAA1598 | 57698 | 10 | 1 |
| KIAA1847 | 84619 | 26 | 1 |
| KIAA1985 | 79628 | 2 | 1 |
| KIAA2026 | 158358 | 73 | 1 |
| KIBRA | 23286 | 27 | 1 |
| KIF21A | 55605 | 79 | 1 |
| KIF3A | 11127 | 177 | 1 |
| KPNA3 | 3839 | 100 | 12 |
| KPNA4 | 3840 | 137 | 1 |
| KPNA6 | 23633 | 6 | 1 |
| KPNB1 | 3837 | 161 | 1 |
| KRT15 | 3866 | 45 | 1 |
| KTN1 | 3895 | 346 | 34 |
| LAMA1 | 284217 | 3 | 1 |
| LAMA4 | 3910 | 105 | 1 |
| LAMB1 | 3912 | 301 | 2 |
| LAMB2 | 3913 | 147 | 1 |
| LAMB3 | 3914 | 77 | 3 |
| LAMC1 | 3915 | 232 | 9 |
| LARP | 23367 | 137 | 9 |
| LATS1 | 9113 | 16 | 1 |
| LDHB | 3945 | 56 | 2 |
| LDOC1 | 23641 | 51 | 44 |
| LEPREL1 | 55214 | 11 | 1 |
| LOC157567 | 157567 | 4 | 2 |
| LOC201176 | 201176 | 27 | 1 |
| LOC221061 | 221061 | 113 | 1 |
| LOC51061 | 51061 | 69 | 5 |
| LOC55580 | 55580 | 159 | 1 |
| LOC92154 | 92154 | 50 | 2 |
| LPL | 4023 | 11 | 1 |
| LRP11 | 84918 | 3 | 1 |
| LRRN6A | 84894 | 40 | 3 |
| LTF | 4057 | 141 | 5 |
| MACF1 | 23499 | 1167 | 410 |
| MADH5 | 4090 | 1 | 1 |
| MAP1A | 4130 | 234 | 2 |
| MAP3K5 | 4217 | 12 | 1 |
| MAP3K8 | 1326 | 3 | 1 |
| MARLIN1 | 152789 | 28 | 7 |
| MAT2B | 27430 | 7 | 1 |
| MATR3 | 9782 | 100 | 1 |
| MBD2 | 8932 | 9 | 1 |
| MDK | 4192 | 4 | 1 |
| MEF2D | 4209 | 33 | 2 |
| MGA | 23269 | 33 | 2 |
| MGC23401 | 196441 | 57 | 11 |
| MGC26717 | 285237 | 7 | 1 |
| MGC35402 | 399669 | 1 | 1 |
| MGC7036 | 196383 | 31 | 1 |
| MIZ1 | 9063 | 45 | 1 |
| MKRN2 | 23609 | 6 | 8 |
| MLLT4 | 4301 | 84 | 1 |
| MPHOSPH10 | 10199 | 151 | 4 |
| MRE11A | 4361 | 5 | 23 |
| MRPL51 | 51258 | 1 | 1 |
| MTATP8 | 4509 | 650 | 1 |
| MTCO1 | 4512 | 1063 | 6 |
| MTIF2 | 4528 | 16 | 12 |
| MTND2 | 4536 | 663 | 3 |
| MTND4 | 4538 | 566 | 8 |
| MTR | 4548 | 7 | 2 |
| MTUS1 | 57509 | 55 | 3 |
| MY048 | 140597 | 40 | 2 |
| MYEF2 | 50804 | 26 | 1 |
| MYH11 | 4629 | 394 | 1 |
| MYH7 | 4625 | 48 | 10 |
| MYH9 | 4627 | 308 | 1 |
| MYL9 | 10398 | 67 | 1 |
| MYO10 | 4651 | 128 | 2 |
| MYO18A | 399687 | 233 | 1 |
| MYO6 | 4646 | 290 | 4 |
| NA | 390718 | 342 | 1 |
| NA | 389314 | 53 | 1 |
| NA | 400320 | 41 | 4 |
| NA | 401483 | 13 | 2 |
| NA | 401002 | 11 | 2 |
| NA | 387671 | 5 | 1 |
| NA | 389347 | 4 | 2 |
| NA | 402535 | 2 | 2 |
| NA | 387762 | 1 | 1 |
| NACSIN | 23301 | 86 | 1 |
| NAP1L3 | 4675 | 16 | 1 |
| NAPB | 63908 | 29 | 3 |
| NCF1 | 4687 | 9 | 1 |
| NCKIPSD | 51517 | 26 | 1 |
| NCOA3 | 8202 | 95 | 1 |
| NCOR1 | 9611 | 120 | 9 |
| NDE1 | 54820 | 32 | 1 |
| NDP52 | 10241 | 218 | 1 |
| NDUFS2 | 4720 | 16 | 1 |
| NDUFS6 | 4726 | 38 | 1 |
| NEFH | 4744 | 150 | 1 |
| NFASC | 23114 | 24 | 1 |
| NFE2L3 | 9603 | 23 | 1 |
| NFKB1 | 4790 | 338 | 9 |
| NKTR | 4820 | 83 | 1 |
| NPIP | 9284 | 16 | 1 |
| NPM1 | 4869 | 156 | 1 |
| NUCB1 | 4924 | 87 | 1 |
| NUCKS | 64710 | 41 | 2 |
| NUMA1 | 4926 | 281 | 6 |
| NUPL1 | 9818 | 101 | 9 |
| NYREN18 | 51667 | 28 | 13 |
| NY-REN-7 | 285596 | 2 | 1 |
| OPTN | 10133 | 137 | 27 |
| OSTF1 | 26578 | 127 | 101 |
| P4HA1 | 5033 | 57 | 25 |
| P4HB | 5034 | 97 | 2 |
| PACSIN1 | 29993 | 110 | 7 |
| PAFAH1B1 | 5048 | 14 | 1 |
| PAI-RBP1 | 26135 | 81 | 1 |
| PAK1IP1 | 55003 | 4 | 1 |
| PCBP2 | 5094 | 88 | 1 |
| PCF11 | 51585 | 53 | 2 |
| PCM1 | 5108 | 244 | 1 |
| PCNT2 | 5116 | 167 | 1 |
| PCQAP | 51586 | 36 | 4 |
| PDCD6IP | 10015 | 141 | 1 |
| PDCD8 | 9131 | 12 | 1 |
| PDE8B | 8622 | 3 | 1 |
| PECAM1 | 5175 | 16 | 1 |
| PEG3 | 5178 | 55 | 1 |
| PFDN5 | 5204 | 80 | 1 |
| PFKP | 5214 | 23 | 1 |
| PGBD4 | 161779 | 9 | 1 |
| PHC1 | 1911 | 15 | 1 |
| PHIP | 55023 | 23 | 1 |
| PIAS3 | 10401 | 80 | 1 |
| PIK3R1 | 5295 | 87 | 133 |
| PIK3R2 | 5296 | 38 | 23 |
| PIK3R3 | 8503 | 43 | 45 |
| PKM2 | 5315 | 72 | 25 |
| PLEC1 | 5339 | 463 | 5 |
| PNLIP | 5406 | 28 | 1 |
| PPA2 | 27068 | 3 | 4 |
| PPARG | 5468 | 151 | 26 |
| PPL | 5493 | 133 | 4 |
| PPP1R12A | 4659 | 74 | 1 |
| PPP3R1 | 5534 | 20 | 1 |
| PRKCB1 | 5579 | 22 | 1 |
| PRKCBP1 | 23613 | 63 | 1 |
| PRKCG | 5582 | 9 | 1 |
| PRNP | 5621 | 53 | 1 |
| PRPF40A/HYPA | 55660 | 222 | 1547 |
| PRSS1 | 5644 | 23 | 2 |
| PS1D | 51538 | 4 | 1 |
| PSMC1 | 5700 | 29 | 1 |
| PSMC2 | 5701 | 23 | 1 |
| PSMC3 | 5702 | 74 | 2 |
| PSMD1 | 5707 | 43 | 1 |
| PSMD2 | 5708 | 43 | 1 |
| PSMD4 | 5710 | 156 | 6 |
| PSMF1 | 9491 | 13 | 1 |
| PTK6 | 5753 | 9 | 5 |
| PTPN18 | 26469 | 2 | 3 |
| PTPNS1 | 140885 | 24 | 1 |
| PTPRE | 5791 | 2 | 1 |
| RANBP2 | 5903 | 266 | 7 |
| RANBP9 | 10048 | 1210 | 4 |
| RARA | 5914 | 19 | 1 |
| RASA1 | 5921 | 95 | 1 |
| RASA3 | 22821 | 5 | 1 |
| RGS1 | 5996 | 11 | 1 |
| RGS12 | 6002 | 20 | 1 |
| RHPN2 | 85415 | 3 | 1 |
| RNASE3L | 29102 | 15 | 1 |
| RNF20 | 56254 | 75 | 3 |
| RNF40 | 9810 | 73 | 6 |
| ROBO3 | 64221 | 2 | 1 |
| ROCK2 | 9475 | 79 | 2 |
| RPIB9 | 154661 | 1 | 1 |
| RPL14 | 9045 | 98 | 1 |
| RPL18A | 6142 | 131 | 2 |
| RPL19 | 6143 | 165 | 3 |
| RPL23 | 9349 | 191 | 1 |
| RPL26 | 6154 | 535 | 6 |
| RPL31 | 6160 | 138 | 2 |
| RPL4 | 6124 | 142 | 3 |
| RPL6 | 6128 | 95 | 1 |
| RPL7A | 6130 | 340 | 3 |
| RPLP2 | 6181 | 17 | 1 |
| RPS13 | 6207 | 64 | 1 |
| RPS15 | 6209 | 128 | 2 |
| RPS15A | 6210 | 46 | 1 |
| RPS2 | 6187 | 47 | 1 |
| RPS20 | 6224 | 752 | 1 |
| RPS23 | 6228 | 36 | 4 |
| RPS27 | 6232 | 329 | 6 |
| RPS7 | 6201 | 114 | 1 |
| RTN4 | 57142 | 265 | 1 |
| S100A9 | 6280 | 85 | 1 |
| SAFB | 6294 | 136 | 1 |
| SAMD4 | 23034 | 41 | 1 |
| SART1 | 9092 | 44 | 1 |
| SASH1 | 23328 | 38 | 8 |
| SCN1B | 6324 | 4 | 1 |
| SDCCAG8 | 10806 | 53 | 2 |
| SEC23IP | 11196 | 26 | 1 |
| SELL | 6402 | 2 | 1 |
| SERPING1 | 710 | 30 | 1 |
| SERTAD1 | 29950 | 50 | 1 |
| SET | 6418 | 117 | 2 |
| SF3B1 | 23451 | 182 | 6 |
| SF3B2 | 10992 | 172 | 1 |
| SFRS10 | 6434 | 17 | 1 |
| SFRS11 | 9295 | 163 | 1 |
| SFRS14 | 10147 | 63 | 1 |
| SFRS8 | 6433 | 59 | 2 |
| SH3YL1 | 26751 | 25 | 1 |
| SLC2A10 | 81031 | 3 | 1 |
| SLC4A1AP | 22950 | 32 | 3 |
| SLC9A1 | 6548 | 843 | 215 |
| SLU7 | 10569 | 95 | 1 |
| SMARCA1 | 6594 | 61 | 1 |
| SMARCA5 | 8467 | 123 | 1 |
| SMARCC1 | 6599 | 73 | 1 |
| SMARCC2 | 6601 | 147 | 1 |
| SMARCE1 | 6605 | 125 | 2 |
| SMG1 | 23049 | 88 | 1 |
| SMYD2 | 56950 | 17 | 2 |
| SNAP25 | 6616 | 43 | 1 |
| SNW1 | 22938 | 94 | 1 |
| SOD1 | 6647 | 22 | 1 |
| SORBS1 | 10580 | 90 | 22 |
| SP1 | 6667 | 20 | 1 |
| SP3 | 6670 | 56 | 9 |
| SP8 | 221833 | 3 | 1 |
| SPARCL1 | 8404 | 508 | 2 |
| SPTAN1 | 6709 | 848 | 114 |
| SPTB | 6710 | 133 | 1 |
| SPTBN1 | 6711 | 1395 | 17 |
| SPTBN2 | 6712 | 317 | 5 |
| SR140 | 23350 | 64 | 1 |
| SR-A1 | 58506 | 48 | 1 |
| SRC | 6714 | 28 | 1 |
| SREBF2 | 6721 | 301 | 19 |
| SRGAP1 | 57522 | 16 | 18 |
| SRGAP2 | 23380 | 29 | 6 |
| SRGAP3 | 9901 | 32 | 9 |
| SRISNF2L | 23132 | 29 | 1 |
| SRPR | 6734 | 17 | 1 |
| SRRM2 | 23524 | 364 | 1 |
| SSFA2 | 6744 | 38 | 2 |
| STARD13 | 90627 | 12 | 1 |
| STK11IP | 114790 | 2 | 1 |
| STS | 412 | 1 | 1 |
| STX7 | 8417 | 4 | 2 |
| SULF1 | 23213 | 20 | 1 |
| SUPT5H | 6829 | 103 | 3 |
| SYNCRIP | 10492 | 70 | 1 |
| SYNE1 | 23345 | 538 | 32 |
| SYNE2 | 23224 | 333 | 43 |
| SYNJ2 | 8871 | 10 | 1 |
| SYNPO2 | 171024 | 61 | 7 |
| TACC1 | 6867 | 59 | 6 |
| TACC2 | 10579 | 41 | 2 |
| TAF1 | 6872 | 569 | 6 |
| TANK | 10010 | 24 | 11 |
| TAO1 | 9344 | 26 | 1 |
| TAZ | 25937 | 16 | 1 |
| TCERG1 | 10915 | 100 | 162 |
| TF | 7018 | 100 | 1 |
| TFAM | 7019 | 9 | 1 |
| THBS2 | 7058 | 19 | 1 |
| TIEG | 7071 | 25 | 1 |
| TIEG2 | 8462 | 12 | 7 |
| TIZ | 171392 | 42 | 316 |
| TJP1 | 7082 | 128 | 1 |
| TLN1 | 7094 | 209 | 3 |
| TLOC1 | 7095 | 71 | 1 |
| TMEM57 | 55219 | 33 | 3 |
| TNFSF10 | 8743 | 9 | 1 |
| TNPO1 | 3842 | 49 | 1 |
| TNR | 7143 | 49 | 1 |
| TNRC11 | 9968 | 21 | 1 |
| TNRC6 | 27327 | 199 | 1 |
| TPM3 | 7170 | 72 | 1 |
| TPT1 | 7178 | 127 | 2 |
| TRAFD1 | 10906 | 10 | 7 |
| TRIM37 | 4591 | 60 | 2 |
| TRIM38 | 10475 | 1 | 1 |
| TRIM41 | 90933 | 15 | 1 |
| TRIO | 7204 | 218 | 1 |
| TRIP11 | 9321 | 180 | 1 |
| TRIP15 | 9318 | 103 | 4 |
| UACA | 55075 | 87 | 2 |
| UBADC1 | 10422 | 71 | 11 |
| UBAP2 | 55833 | 47 | 1 |
| UBC | 7316 | 547 | 2 |
| UBE2E3 | 10477 | 3 | 1 |
| UBE2J1 | 51465 | 1 | 1 |
| UBQLN1 | 29979 | 382 | 26 |
| UBQLN2 | 29978 | 173 | 1 |
| USP9X | 8239 | 77 | 71 |
| UTRN | 7402 | 223 | 6 |
| VIL2 | 7430 | 130 | 6 |
| VIM | 7431 | 198 | 17 |
| VPS39 | 23339 | 24 | 1 |
| WAC | 51322 | 95 | 59 |
| WBP4 | 11193 | 56 | 11 |
| XPO1 | 7514 | 29 | 1 |
| XPO4 | 64328 | 29 | 1 |
| XPO6 | 23214 | 18 | 1 |
| XPO7 | 23039 | 29 | 1 |
| XPOT | 11260 | 35 | 1 |
| YWHAB | 7529 | 203 | 12 |
| YWHAE | 7531 | 358 | 6 |
| ZBTB16 | 7704 | 26 | 3 |
| ZF | 58487 | 5 | 1 |
| ZFH4 | 79776 | 57 | 1 |
| ZFP91 | 80829 | 18 | 1 |
| ZFYVE19 | 84936 | 16 | 5 |
| ZHX1 | 11244 | 35 | 1 |
| ZMYM1 | 79830 | 9 | 2 |
| ZNF133 | 7692 | 8 | 4 |
| ZNF24 | 7572 | 49 | 1 |
| ZNF258 | 9204 | 24 | 1 |
| ZNF286 | 57335 | 12 | 1 |
| ZNF382 | 84911 | 3 | 1 |
| ZNF451 | 26036 | 67 | 3 |
| ZNF537 | 57616 | 17 | 4 |
| ZNF655 | 79027 | 85 | 3 |
| ZNF91 | 7644 | 137 | 7 |
