# Supplementary material for: Huntingtin Interacting Proteins Are Genetic Modifiers of Neurodegeneration
Source: PLoS Genet. 2007 May 11;3(5):e82. doi: 10.1371/journal.pgen.0030082 (PMC1866352; doi:10.1371/journal.pgen.0030082)
Supplement: Supplementary file 6 [file pgen.0030082.st004.doc]

| **Human Gene Symbol (NCBI)** | **Human Protein Symbol (ExPASy)** | **Method** | **Fly Gene Symbol** | **Flybase ID** | **expect value** | **Modifier** |
| --- | --- | --- | --- | --- | --- | --- |
| ADD3 | ADDG_HUMAN | Y2H | hts | FBgn0004873 | -99 | Yes |
| AP2A1; AP2A2 | AP2A1_HUMAN; AP2A2_HUMAN | Y2H | a-Adaptin | FBgn0015567 | 0 | Yes |
| API5 | API5_HUMAN | Y2H | Aac11 | FBgn0027885 | -115 | Yes |
| ARS2 | ARS2_HUMAN | Y2H | CG7843 | FBgn0033062 | -98 | Yes |
| BAZ1A | BAZ1A_HUMAN | Y2H | acf1 | FBgn0027620 | -42 | No |
| BZW2 | Q9Y6E2_HUMAN | Y2H | eIF-5C | FBgn0037346 | -119 | Yes |
| CTNNB1 | CTNB1_HUMAN | Y2H | armadillo | FBgn0000117 | 0 | Yes |
| CUL2 | CUL2_HUMAN | Y2H | cul-2 | FBgn0032956 | 0 | Yes |
| DNAJA5 | Q86VC6_HUMAN | Y2H | CG2790 | FBgn0027599 | -76 | Yes |
| DNCH1 | CYHC_HUMAN | Y2H | Dhc64C | FBgn0010349 | 0 | Yes |
| DNM1 | DYN1_HUMAN | Y2H | shibire | FBgn0003392 | 0 | Yes |
| FEZ1 | FEZ1_HUMAN | Y2H | Unc-76 | FBgn0040395 | -26 | Yes |
| FLJ13386/Cep36 | CEP63_HUMAN | Y2H | zipper | FBgn0005634 | -18 | Yes |
| SRGAP1 | SRGP1_HUMAN | Y2H | RhoGAP92B | FBgn0038747 | -18 | Yes |
| HIP2 | UBC1_HUMAN | Y2H | UbcD4 | FBgn0015321 | -70 | Yes |
| KIAA1229 | Q9ULJ1_HUMAN | Y2H | CLIP-190 | FBgn0020503 | -12 | Yes |
| MEF2D | MEF2D_HUMAN | Y2H | Mef2 | FBgn0011656 | -47 | Yes |
| NCOR1 | NCOR1_HUMAN | Y2H | Smrter | FBgn0024308 | -48 | Yes |
| PACSIN1 | PACN1_HUMAN | Y2H | Syndapin | FBgn0053094 | -87 | Yes |
| PPA2 | IPYR2_HUMAN | Y2H | nurf-38 | FBgn0016687 | -72 | No |
| PPARG | PPAT_HUMAN | Y2H | Eip75B | FBgn0000568 | -53 | Yes |
| PPL | PEPL_HUMAN | Y2H | short stop | FBgn0013733 | -54 | Yes |
| PRKCBP1 | PKCB1_HUMAN | Y2H | CG1815 | FBgn0039863 | -63 | Yes |
| PTK6 | PTK6_HUMAN | Y2H | Src42A | FBgn0029082 | -10 | Yes |
| ROCK2 | ROCK2_HUMAN | Y2H | rok | FBgn0051163 | -14 | No |
| SORBS1 | SRBS1_HUMAN | Y2H | CAP | FBgn0033504 | -51 | Yes |
| SRGAP2 | FNBP2_HUMAN | Y2H | CdGAPr | FBgn0035827 | -14 | Yes |
| SUPT5H | O00267_HUMAN | Y2H | Spt5 | FBgn0040273 | 0 | Yes |
| TACC1 | TACC1_HUMAN | Y2H | tacc | FBgn0026620 | -22 | No |
| USP9X | USP9X_HUMAN | Y2H | fat facets | FBgn0005632 | 0 | Yes |
| ZBTB16 | ZBT16_HUMAN | Y2H | CG12299 | FBgn0032295 | -43 | No |
| ZNF91 | ZNF91_HUMAN | Y2H | crooked legs | FBgn0020309 | -118 | Yes |
| CLTC | CLH1_HUMAN | MS/Y2H | Chc | FBgn0000319 | 0 | Yes |
| GAPDH | G3P1_HUMAN | MS/Y2H | Gpdh | FBgn0001091 | -147 | Yes |
| YWHAB | 1433B_HUMAN | MS/Y2H | 14-3-3zeta | FBgn0004907 | -109 | Yes |
| ALDOC | ALDOC_HUMAN | MS | aldolase | FBgn0000064 | -129 | No |
| ASPH | ASPH_HUMAN | MS | Asph | FBgn0034075 | -104 | Yes |
| ATP6V0E | VA0H_HUMAN | MS | L(1)G0030 | FBgn0026708 | 0 | Yes |
| CACNA2D1 | CAC2D_HUMAN | MS | CG12455 | FBgn0028859 | -85 | Yes |
| CDH13 | CAD13_HUMAN | MS | Cadherin-N | FBgn0015609 | -51 | Yes |
| COX6C | COX6C_HUMAN | MS | cyclope | FBgn0015031 | -10 | Yes |
| CPT1B | CPT1B_HUMAN | MS | CPT1 | FBgn0027842 | 0 | Yes |
| CYC1 | CY1_HUMAN | MS | CG4769 | FBgn0035600 | -87 | No |
| ENO1/ENO3 | ENOA_HUMAN;  ENOB_HUMAN | MS | enolase | FBgn0000579 | -177 | No |
| EPB41L1 | E41L1_HUMAN | MS | coracle | FBgn0010434 | -122 | Yes |
| FBN2 | FBN2_HUMAN | MS | dumpy | FBgn0053196 | -157 | No |
| GFAP | GFAP_HUMAN | MS | Lamin C | FBgn0010397 | -40 | Yes |
| GNAZ | GNAZ_HUMAN | MS | G-ia65A | FBgn0001104 | -118 | Yes |
| GNB2 | GBB2_HUMAN | MS | Gbeta13F | FBgn0001105 | -165 | Yes |
| GPM6a | GPM6A_HUMAN | MS | M6 | FBgn0037092 | -23 | Yes |
| ITPR1 | ITPR1_HUMAN | MS | Itp-r83A | FBgn0010051 | 0 | Yes |
| NDUFA9 | NUEM_HUMAN | MS | CG6020 | FBgn0037001 | -63 | No |
| NDUFB10 | NIDM_HUMAN | MS | Pdsw | FBgn0021967 | -16 | Yes |
| NDUFV1 | NUBM_HUMAN | MS | CG9140 | FBgn0031771 | 0 | Yes |
| NEGR1 | NEGR1_HUMAN | MS | Lachesin | FBgn0010238 | -28 | Yes |
| PHB2 | PHB2_HUMAN | MS | L(2)03709 | FBgn0010551 | -81 | No |
| SEPT7 (CDC10) | SEPT7_HUMAN | MS | peanut | FBgn0014879 | -75 | Yes |
| STX1A | STX1A_HUMAN | MS | Syntaxin 1A | FBgn0013343 | -97 | Yes |
| TPI1 | TPIS_HUMAN | MS | tpi | FBgn0003721 | -59 | No |
| VDAC2 | VDAC2_HUMAN | MS | porin | FBgn0004363 | -97 | Yes |
