# Supplementary material for: Huntingtin Interacting Proteins Are Genetic Modifiers of Neurodegeneration
Source: PLoS Genet. 2007 May 11;3(5):e82. doi: 10.1371/journal.pgen.0030082 (PMC1866352; doi:10.1371/journal.pgen.0030082)
Supplement: Supplementary file 7 [file pgen.0030082.st005.doc]

| **Human Gene symbol** | **Source** | ***Drosophila* ortholog** | **Function** | **Over expression allele(s)** | **E/S** | **Loss of function allele(s)** | **E/S** |
| --- | --- | --- | --- | --- | --- | --- | --- |
| Cytoskeletal organization and biogenesis | | | | | | | |
| DNCI2* | MS | *sw* | Structural constituent | NA | NA | 1 | E |
| DNM1 | Y2H | *shibire* | Intracellular protein transport, structural constituent of the cytoskeleton | NA | NA | 1 | E |
| TLN1* | Y2H | *rhea* | Cytoskeletal anchoring | NA | NA | 1 | E |
| Signal transduction | | | | | | | |
| CDH13 | MS | *Cadherin-N* | Axon guidance, signal transduction | NA | NA | M12 | S |
| MLLT4* | Y2H | *canoe* | JNK cascade, Ras protein signal transduction | NA | NA | 2 | S |
| SRGAP1 | Y2H | *RhoGap92B* | GTPase activator, intracellular protein transport | EY04974 | S | NA | NA |
| SRGAP2 | Y2H | *CdGAPr* | GTPase activator activity, intracellular signalling cascade | NA | NA | P{UAS-CdGAPr-dsRNA}3.1 | S |
| TRIO* | Y2H | *Trio* | G-protein coupled receptor signaling pathway | KG606642 | E | NA | NA |
| EPB41L1 | MS | *coracle* | Cytoskeletal protein binding, structural constituent of the cytoskeleton | NA | NA | k08713 | S |
|  |  |  |  |  |  |  |  |
| GNB2 | MS | *Gβ13F* | G-protein coupled receptor signaling pathway | EP1071 | E | NA | NA |
| Synaptic transmission | | | | | | | |
| AP2A1; AP2A2 | Y2H | *α-Adaptin* | Intracellular protein transport, neurotransmitter secretion | NA | NA | 06694 | S |
| PACSIN1 | Y2H/LIT | *Syndapin* | Intracellular signalling cascade, neurotransmitter secretion | EP877 | S | NA | NA |
| ATP6V0E | MS | *L(1)G0030* | Lyase activity | G00300 | E | NA | NA |
| VAMP2* | MS | *syb* | Neurotransmitter secretion | K07703 | S | NA | NA |
| Proteolysis/peptidolysis or ubiquitin cycle | | | | | | | |
| CUL2 | Y2H | *cul-2* | Proteolysis and peptidolysis | EY91245 | S | NA | NA |
| DNAJA5 | Y2H | *CG2790* | Protein folding | EP412 | E | NA | NA |
| PRKCBP1 | Y2H | *CG1815* | Protein ubiquitination, signal transduction | EY04635 | S | NA | NA |
| HIP2 | Y2H/LIT | *UbcD4* | Ubiquitin conjugating enzyme activity | EY05497 | S | NA | NA |
| Regulation of transcription or translation | | | | | | | |
| BZW2 | Y2H | *eIF-5C* | Translational regulation | EY11022 | E | NA | NA |
| NCOR1 | Y2H/LIT | *Smrter* | Regulation of transcription from Pol II promoter | NA | NA | BG01648 | S |
| SUPT5H | Y2H | *Spt5* | RNA elongation from Pol II promoter | NA | NA | MGE-3 | E |
| CRSP3* | MS | *Med23* | Regulation of transcription from PolII promoter | KG00948 | S | NA | NA |
| TAF4* | Y2H | *Taf4* | Transcription initiation from PolII promoter | 1 | E | NA | NA |
| Other | | | | | | | |
| API5 | Y2H | *Aac11* | Anti-apoptosis | NA | NA | k06710 | S |
| ARS2 | Y2H | *CG7843* | Defense response | NA | NA | KG07177 | S |
| NDUFV1 | MS | *CG9140* | Mitochondrial electron transport | NA | NA | KG01287 | E |
| CPT1B | MS | *CPT1* | Amino acid metabolism | NA | NA | KG01596 | E |
| COX6C | MS | *cyclope* | Mitochondrial electron transport | NA | NA | e03803 | E |
| BMP2K* | Y2H | *Nak* | ATP binding, protein serine/threonine kinase activity | UAS:Nak^15^ | S | NA | NA |
